# Circadian Rhythm Disruption Exacerbates Autoimmune Uveitis: The Essential Role of PER1 in Treg Cell Metabolic Support for Stability and Function

**DOI:** 10.1002/advs.202400004

**Published:** 2025-01-17

**Authors:** Wenjie Zhu, Guanyu Chen, Zhiqiang Xiao, Minzhen Wang, Zhuang Li, Yuxun Shi, Xiaohui Luo, Zuoyi Li, Haixiang Huang, Xiaoqing Chen, Lingyi Liang, Dan Liang

**Affiliations:** ^1^ State Key Laboratory of Ophthalmology Zhongshan Ophthalmic Center Sun Yat‐sen University Guangdong Provincial Key Laboratory of Ophthalmology and Visual Science Guangzhou 510060 China

**Keywords:** autoimmune uveitis, cell metabolism, circadian rhythm, PER1, Treg

## Abstract

Circadian rhythm plays a critical role in the progression of autoimmune diseases. While our previous study demonstrated the therapeutic effects of melatonin in experimental autoimmune uveitis, the involvement of circadian rhythm remained unclear. Using a light‐induced circadian rhythm disruption model, we showed that disrupted circadian rhythms exacerbate autoimmune uveitis by impairing the stability and function of Treg cells. Mechanistically, we identified the core clock gene *Per1*, which is significantly reduced under circadian disruption, is essential for Treg cell metabolism and immunoregulatory function. This study underscores the pivotal role of circadian rhythm‐related Treg cells in autoimmune disease progression.

## Graphical abstract

Circadian rhythms emerge as significant factors in the progression of autoimmune diseases. However, whether and how circadian rhythms are involved remains uncovered. Using a light‐induced rhythm‐disrupting model, it is demonstrated that circadian rhythm disruption aggravates autoimmune uveitis due to Treg impairment. Mechanically, the core‐clock gene, *Per1*, is identified as essential for Treg metabolism and function. This study highlights the importance of circadian rhythm‐related Treg in autoimmune disease.

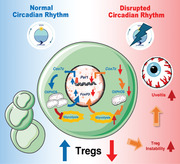

## 1 Introduction

The Earth's daily rotation, creating 24‐h environmental cycles, has been pivotal in shaping circadian rhythms across living organisms. In mammals, these rhythms synchronize with the environment through exposure to the light‐dark cycle, orchestrated by a neural pacemaker in the hypothalamic suprachiasmatic nuclei (SCN) that directly responds to light.^[^ ^1^ ^]^ These circadian rhythms are directed by autonomous biological clocks within cells.^[^ ^2^ ^]^ Decades of extensive research have unveiled a set of conserved transcription factors operating within intricate transcription‐translation feedback loops. Notable members including circadian locomotor output cycles protein kaput (CLOCK) and brain and muscle ARNT‐like 1 (BMAL1) proteins form complexes that regulate target genes through E‐box elements. Additionally, circadian transcription factors such as period circadian protein homolog 1 (PER1), PER2, cryptochrome 1 (CRY1), CRY2, REV‐ERBα (NR1D1), REV‐ERBβ (NR1D2), chondrocytes protein 1 (also known as BHLHE40), and DEC2 primarily function as repressors. The orchestrated negative feedback, governed mainly by PER and CRY proteins, briefly suppresses CLOCK‐BMAL1 complexes on target genes. This repression is alleviated at night, resulting in the observed oscillations.^[^ ^3^ ^]^

Over the past decade, circadian rhythms have emerged as significant factors in the development and progression of autoimmune diseases.^[^ ^4^ ^]^ Genome‐wide association studies have uncovered potential links between core‐clock gene polymorphisms and susceptibility to inflammatory bowel disease,^[^ ^5^ ^]^ asthma,^[^ ^6^ ^]^ and multiple sclerosis (MS).^[^ ^7^ ^]^ Clinical studies have confirmed associations between night shift work and the incidence of inflammatory arthritis (RA),^[^ ^8^ ^]^ asthma,^[^ ^9^ ^]^ and MS.^[^ ^10^ ^]^ Mouse model research has shown that environmental manipulation or perturbation in core‐clock genes exacerbates disease severity^[^ ^11^ ^]^ or disease rhythmicity.^[^ ^12^ ^]^ Notably, in experimental autoimmune encephalomyelitis (EAE) mice, a mouse model of MS, the absence of REV‐ERBα leads to increased RORγt+ cell frequency in the central nervous system and a reduction in anti‐inflammatory regulatory T cells (Tregs).^[^ ^13^ ^]^ These findings highlight the circadian clock's influence on immune tolerance, especially through CD4+ T cells.

Autoimmune uveitis is an eye condition characterized by inflammatory damage to the retina and choroid, primarily instigated by autoimmune T cells.^[^ ^14^ ^]^ While there is a paucity of human data directly linking circadian disturbance with increased incidence or severity of uveitis, various studies have noted that uveitis patients often experience poor‐quality sleep and shorter sleep duration compared to healthy individuals.^[^ ^15^ ^]^ Our prior research demonstrated the therapeutic potential of melatonin, a key hormone for regulating sleep and circadian rhythms, in mitigating the severity of the disease in experimental autoimmune uveitis (EAU) mice.^[^ ^16^ ^]^ Furthermore, a recent report revealed that sleep deprivation exacerbated clinical grades in EAU mice and increased pathogenicity in their Th17 cells.^[^ ^17^ ^]^ Disturbed circadian rhythms may aggravate uveitis by CD4+T cells.

In recent years, the intersection of circadian rhythms and cellular metabolism has gained significant attention.^[^ ^18^ ^]^ Rev‐erbα, a crucial component within the circadian core clock, functions as a heme sensor, orchestrating the cellular clock, glucose homeostasis, and energy metabolism.^[^ ^19^ ^]^ Additionally, a recent study has indicated that seasonal variations in light exposure can impact energy metabolism.^[^ ^20^ ^]^ However, there has been limited investigation into the role of other clock genes in cellular metabolism.

In this study, we investigate whether and how circadian rhythm disruption affects autoimmune uveitis. Using a light‐induced circadian rhythm‐disrupting model, we demonstrate that circadian rhythm regulates Treg cell stability and immunoregulation function via the core‐clock gene *Per1*. We further uncover that *Per1* can modulate Treg cell function through Cytochrome c oxidase 7C (Cox7c)‐related mitochondrial phosphate oxidation. These findings enhance our understanding of how the circadian clock regulates autoimmune uveitis through Treg cells in a Per1‐dependent manner.

## 2 Results

### 2.1 Diurnal Rhythms of CD4+ T Cells and Core‐Clock Genes in Naive and EAU Mice

Previously, literature has detailed the dynamic regulation of clock activity in T cells.^[^ ^21^, ^22^ ^]^ Despite the lack of conclusive evidence for a functional clock within naïve CD4 T cells,^[^ ^23^ ^]^ components of the molecular clock (e.g., Bmal1 or REV‐ERB alpha) have been shown to affect autoreactive T cell function and Th17 cell function.^[^ ^13^, ^24^ ^]^ However, whether a functional cell‐intrinsic circadian clock exists in Tregs remains controversial.^[^ ^25^, ^26^ ^]^

To explore whether there are circadian oscillations of cells in uveitis, we first investigated diurnal variation profiles of eye‐draining lymphocytes of naïve mice and EAU mice, a characterized mouse model of human uveitis. Using flow cytometry, we initially assessed the diurnal variation profiles of different lymphocyte populations. Plotting cell population against time, with Zeitgeber Time (ZT) 0 representing the onset of the light phase and ZT12 marking the dark phase (Figure S1A, Supporting Information). We observed significant variations in the percentages of CD8+ cells in naive mice and diurnal variations in the CD3+ and CD4+ T cells in EAU mice (Figure S1B, Supporting Information).

**Figure 1:**
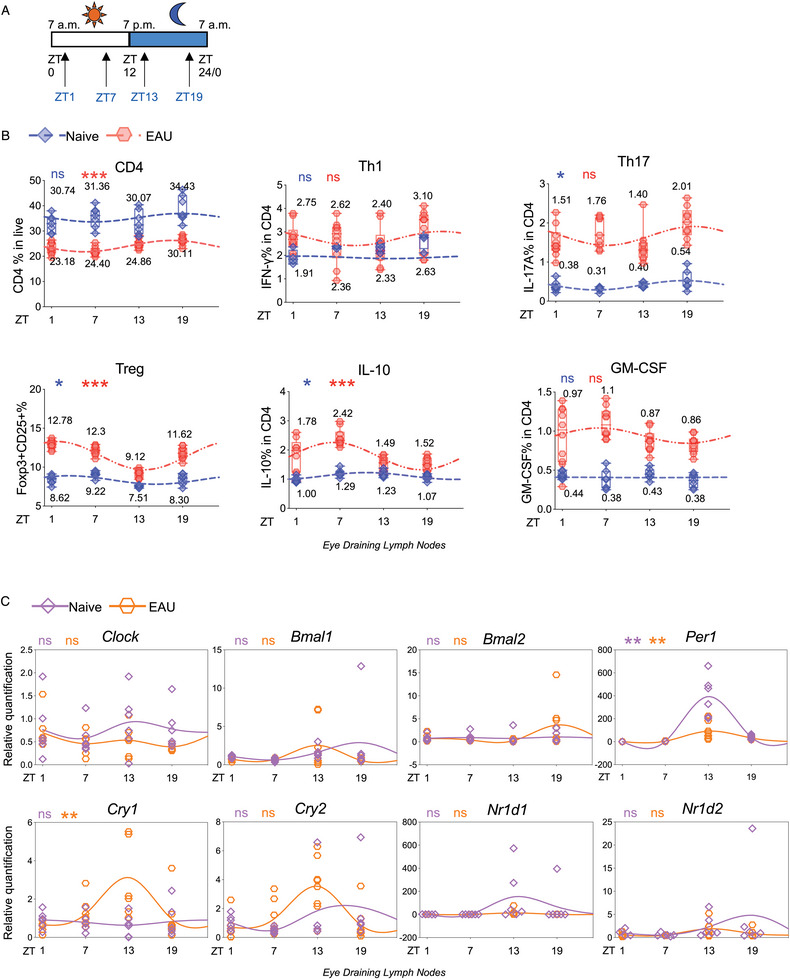
Diurnal rhythms of CD4+ T cells and core‐clock genes in naive and EAU mice. A) Schematic diagram of Zeitgeber Time and the time points for sacrifice mice (ZT1, ZT7, ZT13, ZT19). B) Plots display the percentages of indicated cell subsets (CD4+, Th1, Th17, Treg, IL‐10+ and GM‐CSF+ CD4+ cells) at different time points and their fitted cosine curve in the eye‐draining lymph cells of naïve and EAU mice (21 days post‐immunization). N = 6–10. Data was combined from two experiments. C) A series of core‐clock gene expressions in CD4+ T cells from eye‐draining lymph nodes of naïve and EAU mice were examined by RT‐qPCR at different ZT times. The relative quantification was normalized by the average expressions of ZT1 samples from naïve mice. N = 6–8. Data was combined from two experiments. B,C) Cosine similarity analysis and statistical significance were determined by Cosinor. *p<0.05, **p<0.01, ***p < 0.001, ****p < 0.0001.

Given the pivotal role of CD4+ T cells in autoimmune diseases,^[^ ^27^ ^]^ we further investigated the rhythmicity of CD4+ T cells. We analyzed the CD4+ population and its principal subsets at various Zeitgeber times (ZT1, ZT7, ZT13, and ZT19). CD4+ T cells exhibited rhythmicity specifically in EAU mice, but not in naïve mice. Furthermore, when we dissected CD4+ T cell subsets, we found that only FOXP3+CD25+ Tregs and IL‐10+ CD4+ T cells displayed rhythmicity. Notably, rhythmicity was not observed in Th1 or GM‐CSF+ T cells in either naïve or EAU mice, and Th17 cells exhibited significant rhythmic variation solely in naïve mice (**Figure** **1**A,B).

In addition to immune cells, we investigated the rhythmicity of core‐clock gene expressions in CD4+ T cells extracted from eye‐draining lymph nodes of both naïve and EAU mice. Plots revealed that *Cry1* exhibited significant rhythmicity in CD4+ T cells from EAU mice, and *Per1* exhibited rhythmic oscillations in CD4+ T cells from both naïve and EAU mice (Figure 1C). These findings demonstrated the rhythmic oscillations of CD4+ T cells and core‐clock genes in EAU mice, shedding light on the temporal dynamics of immune responses in autoimmune uveitis.

### 2.2 Circadian Rhythm Disruption Exacerbates EAU

**Figure 2:**
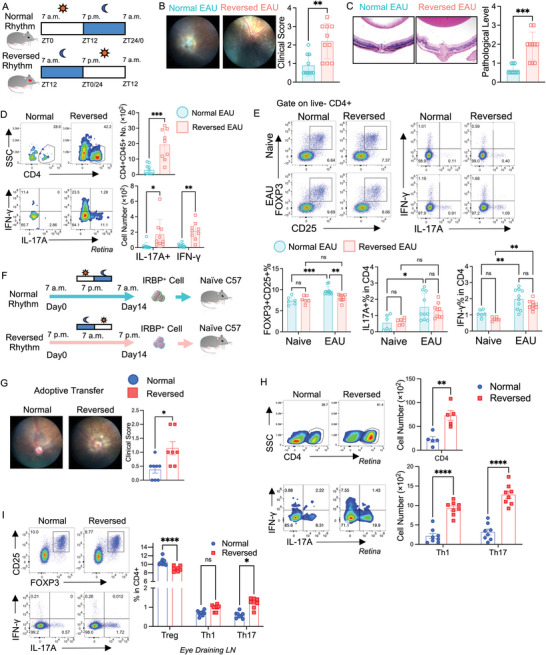
Circadian rhythm disruption exacerbates EAU. A–E) C57BL/6J mice were randomly divided and treated with normal (lights on at 7 a.m., lights off at 7 p.m.) or reversed (lights on at 7 p.m., lights off at 7 a.m.) cycles of light exposure. Part of the mice from each group were immunized with hIRBP1‐20 on Day 0, and mice were sacrificed at ZT1 on Day 21. A) Schematic diagram of the two groups' normal and reversed 12–12 light‐dark cycles. B) Representative images showing the fundus images from the Normal Group and the Reversed Group of EAU mice on Day 20 after immunization. Bar graph showed the clinical scores of those EAU mice. N = 10. Data was combined from three experiments. C) Representative images of eyeballs with HE staining of EAU mice from the Normal Group and the Reversed Group. Bar graph presenting the pathological levels of those EAU mice. N = 10. Data was combined from three experiments. D) Representative FACS plots and bar graphs depicting the cell number of CD45+CD4+ cells, Th1 (CD4+ IFN‐γ+) cells, and Th17 (CD4+ IL‐17A+) cells from the retina of the Normal and the Reversed EAU mice on Day 21 after immunization. N = 9–10. Data was combined from three experiments. E) Representative FACS plots and bar graphs presenting the percentage of Tregs (CD4+CD25+FOXP3+), Th1 cells, and Th17 cells from the eye‐draining lymph nodes of Normal and Reversed EAU mice on Day 21 after immunization. N = 6–10. Data was combined from two experiments. F–I) Adoptive transfer assay: Total lymphocytes from eye‐draining lymph nodes from the Normal and the Reversed EAU mice were stimulated with hIRBP_1‐20_ for 72 h. 1 × 10^7^ of lived suspending cells were transferred back to naïve mice. N = 8. Data was combined from two experiments. F) Schematic diagram of the adoptive transfer assay. G) Representative images of fundoscopy displaying retinal inflammation, and bar plot presenting clinical scores of mice received cells from the Normal or Reversed Group on Day 11 after the transfer. H) Representative FACS plots and bar graphs depicting the cell number of CD45+CD4+ cells, Th1 (CD4+ IFN‐γ+) cells, and Th17 (CD4+ IL‐17A+) cells from the retina of the Normal and the Reversed Adoptive Transfer EAU mice on Day 11 after immunization. I) Representative FACS plots and bar graph presenting the percentage of Tregs (CD4+CD25+FOXP3+), Th1 cells, and Th17 cells from the eye‐draining lymph nodes of Normal and Reversed Adoptive Transfer EAU mice on Day 11 after immunization. Statistical significance was determined by Welch's *t*‐test in (B,C,G), the upper box of (D,H). Statistical significance was determined by Statistical significance was determined by two‐way ANOVA followed by Bonferroni test in the lower box of (D,H), (E,I). Data are presented as mean ± SEM. *p<0.05, **p<0.01, ***p<0.001, ****p<0.0001.

Circadian rhythms are vital biological processes influenced by various environmental factors such as shiftwork, artificial lighting, and jet lag.^[^ ^28^ ^]^ To mimic circadian rhythm disruption, we implemented an irregular light‐dark cycle. We randomly divided mice into two groups. The first group adhered to a conventional 12–12 light‐dark cycle (lights on at 7 a.m., lights off at 7 p.m., referred to as the “Normal Group”). The second group of mice followed a reversed 12‐12 light‐dark cycle (lights on at 7 p.m., lights off at 7 a.m., referred to as the “Reversed Group”) (**Figure** **2**A). By tracking the animals' path, we observed a disrupted travel rhythm, demonstrating that the mice did not fully adapt to the reversed lighting until day 20. Thus, the animals experienced a significant period where their activity rhythms were misaligned to the lighting schedule (Figure S2A, Supporting Information).

On the 21st day following EAU immunization, we observed a significant escalation in both clinical scores (Figure 2B) and pathological severity in the Reversed Group (Figure 2C). Within this group, there was a notable increase in the infiltration of CD4+ T cells in the retina, particularly of the Th1 and Th17 subsets (Figure 2D). However, when examining the eye‐draining lymph nodes, we detected a diminished population of Treg cells (CD25+FOXP3+ cells in the CD4+ population) in Reversed Group EAU mice, with no significant alterations in Th1 or Th17 cell counts compared to their counterparts in the Normal Group EAU mice (Figure 2E, Figure S2D, Supporting Information). Additionally, IL‐10‐producing CD4+ T cells^[^ ^29^ ^]^ and GM‐CSF+ Teffs^[^ ^30^ ^]^ are crucial participants in autoimmune inflammation, and we therefore checked their populations. Eye‐draining lymph nodes of the Reversed Group EAU mice exhibited a markedly decreased fraction of IL‐10‐producing CD4+ T cells and a notable increase in GM‐CSF production from CD4+ T cells when compared to the Normal Group EAU mice (Figure S2B, Supporting Information). In the spleen, we detected a similar phenotype change in the Reversed Group of EAU mice compared to the Normal Group (Figure S2C, Supporting Information).

We conducted an adoptive transfer experiment to substantiate that the higher severity of EAU in the Reversed Group was primarily attributed to T cells (Figure 2F). Fundoscopy revealed that lymphocytes stimulated by hIRPB from the Reversed Group of EAU mice induced more severe clinical manifestations than the Normal Group (Figure 2G). Flow cytometry data revealed a notable increase in the infiltration of CD4+ T cells in the retina of the Reversed Group, along with elevated levels of Th1 and Th17 subsets (Figure 2H). In the eye‐draining lymph nodes, there was a significant decrease in the Treg population and an increase in the Th17 population in the Reversed Group, but not in the Th1 population (Figure 2I).

We are curious whether restoring the reversed light cycle helps attenuate EAU and rescue the decreased Treg population in the Reversed Group. Thus, we divided mice into 3 groups: Normal, Reversed, or Restored (Reversed lighting for 10 days, and restored lighting for the remaining 11 days) cycles of light exposure, and all mice were immunized for EAU. After 21 days, the fundus image showed the restored light cycle did not decrease the clinical score compared to the Reversed Group (Figure S2E, Supporting Information). We saw a significant increase of infiltrating CD4+ T cells in the retina of the Restored Group compared to the Reversed Group with more Th1 and Th17 cells (Figure S2F, Supporting Information). These data indicated a more frequent light rhythm disruption would aggravate inflammation in EAU.

### 2.3 Impairment of Treg Stability and Function due to Circadian Rhythm Disruption

**Figure 3:**
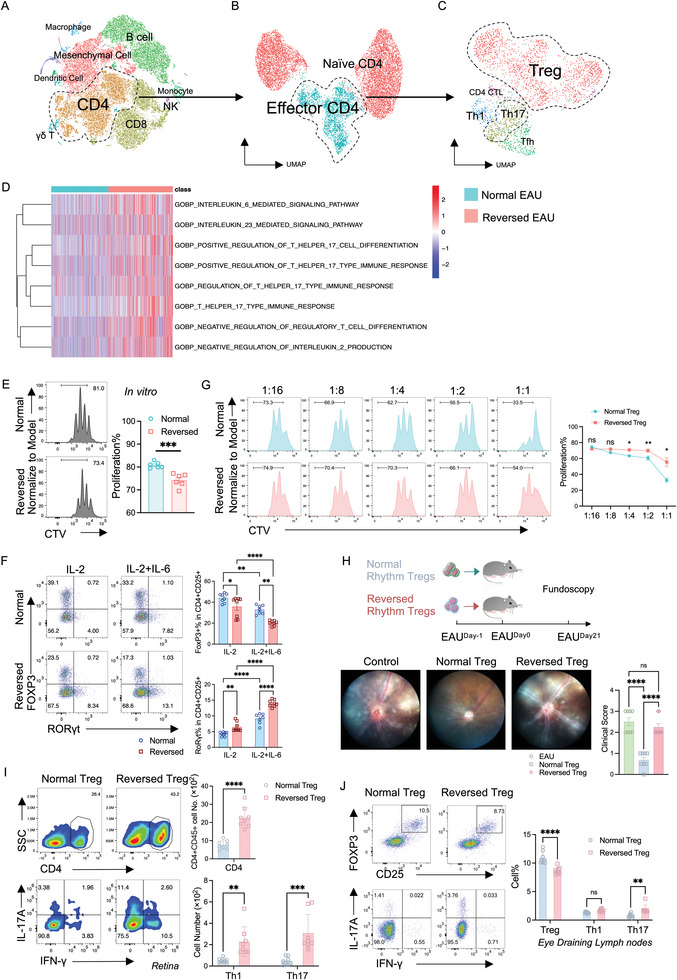
Impairment of Treg stability and function due to circadian rhythm disruption. A–D) Single‐cell RNA sequencing was performed from the eye‐draining lymph nodes of the EAU mice from the Normal Group and the Reversed Group. A) T‐SNE plot presenting the nine cell subsets from scRNA‐seq. B) UMAP projection plot showing the sub‐clusters of CD4+T cells from scRNA‐seq. C) UMAP projection plot showing the sub‐clusters of effector CD4+T cells (Teff) from scRNA‐seq. D) Heatmap depicting the Th17‐related pathways in Tregs between the Normal and the Reversed Group of EAU mice from GSVA analysis of scRNA‐seq. E–G) Mice were treated with normal (lights on at 7 a.m., lights off at 7 p.m.) or reversed (lights on at 7 p.m., lights off at 7 a.m.) cycles of light exposure for 21 days before being sacrificed for in vivo experiments. E) Representative FACS histograms and bar graph displaying the proliferation rate of Cell Trace Violet (CTV) labeled natural Treg cells (CD4+ CD25+) from naïve mice of the Normal and the Reversed Groups. N = 6. Data were combined from two experiments. F) Representative FACS plots and bar graphs depicting the stability of Treg cells (CD4+ CD25+ FOXP3+) isolated from the Normal and the Reversed Groups. The isolated Treg cells were incubated with anti‐CD3/CD28 beads and incubated with IL‐2 (10 ng mL^−1^) with or without IL‐6 (50 ng mL^−1^) for 72 h. N = 7–8. Data were combined from two experiments. (G) Representative FACS histograms and bar graph showing the proliferation rate of Cell Trace Violet (CTV) labeled conventional T co‐cultured with natural Tregs (CD4+ CD25+) isolated from naïve mice of the Normal and the Reversed Groups at various Treg/Tcon cell ratios. N = 6. Data were combined from two experiments. H–J) EAU mice were transferred with 3 × 10^6^ Treg cells (CD4+ CD25+) from naïve mice of the Normal and the Reversed Groups. H) Representative images of fundoscopy presenting the retinal inflammation and the bar graph displaying the clinical score of mice transferred with Treg cells from the Normal and the Reversed groups. N = 8. Data were combined from two experiments. I) Representative FACS plots and bar graphs depicting the cell number of CD45+CD4+ cells, Th1 (CD4+ IFN‐γ+) cells, and Th17 (CD4+ IL‐17A+) cells from the retina of the normal and reversed Treg transfer group on Day 21 after immunization. N = 8. Data was combined from two experiments. J) Representative FACS plots and bar graphs depicting the percentage of Tregs, Th1 cells, and Th17 cells from the eye‐draining lymph nodes of the normal and reversed Treg transfer group on Day 21 after immunization. N = 8. Data was combined from two experiments. Statistical significance was determined by Welch's *t*‐test in (E), upper box of (I). Statistical significance was determined using two‐way ANOVA followed by the Bonferroni test in (G–J). Data presented as mean ± SEM, with *p < 0.05, **p < 0.01, ***p < 0.001, ****p < 0.0001.

To delve into the potential mechanism, we conducted single‐cell (sc) RNA‐seq analysis on eye‐draining lymph nodes from both the Normal and Reversed groups of EAU mice (Figure S3A, Supporting Information). The sc‐RNA sequencing unveiled nine primary cell types, including mesenchymal cells, CD4+ T cells, CD8+ T cells, dendritic cells, B cells, γδ T cells, monocytes, macrophages, and NK cells (**Figure** **3**A). Among CD4+ T cells, two principal subsets emerged: naïve CD4 T cells and effector T cells (Figure 3B). Additionally, there is a significant reduction in Treg cell percentage in the light‐induced Reversed Group in Figure 2E. Consequently, we focus on Treg cell function (Figure 3C).

The Reversed Treg exhibited hallmarks associated with Th17 cells and a higher expression of inflammatory genes (Figure 3D, Figure S3B, Supporting Information). Additionally, we discerned a series of reduced expression of Treg function markers by flow cytometry, including CTLA4, ICOS, CD39, and Nrp‐1 (Figure S3C, Supporting Information). Furthermore, a pronounced upregulation of Th17‐related markers, such as CCR6 and RORγt, was evident in the Reversed Treg (Figure S3D,E, Supporting Information), which indicated the decreased stability of this population.^[^ ^31^ ^]^ Notably, Treg cells from the Reversed Group exhibited impaired proliferation both in vivo and in vitro (Figure 3E, Figure S3F, Supporting Information). In vivo, there is a slight increase in apoptosis compared to Treg cells from the Normal Group (Figure S3G, Supporting Information).

To further substantiate the impaired Treg stability in rhythm‐disrupted Treg cells, we introduced IL‐6 to the Treg stability test. Treg cells were isolated from naïve mice under normal or reversed light exposure conditions, treated with or without IL‐6. Treg cells from the Reversed Group exhibited a significant decrease in FOXP3 percentage and an increase in RORγt percentage compared to the Normal Group (Figure 3F).

To ascertain the functional implications, we isolated Tregs from the Normal and Reversed Groups and conducted function assays in vitro and in vivo. Tregs from the Reversed Group displayed decreased conventional T cell proliferation suppression in in vitro co‐culture experiments (Figure 3G). In vivo, the Reversed Tregs failed to impede the progression of EAU, while Tregs from the Normal Group succeeded (Figure 3H). There was a significant increase in infiltrating CD4+ T cells in the retina of the Reversed Tregs compared to the Normal Tregs, characterized by increased Th1 and Th17 cells (Figure 3I). In eye‐draining lymph nodes, there was a significant decrease in Treg and an increase in Th17 cells (Figure 3J).

These findings collectively revealed the detrimental impact of circadian rhythm disruption on Treg stability and function, unveiling critical insights into the mechanistic basis of immune dysregulation.

### 2.4 Circadian Rhythm Regulation of Treg Function via PER1

**Figure 4:**
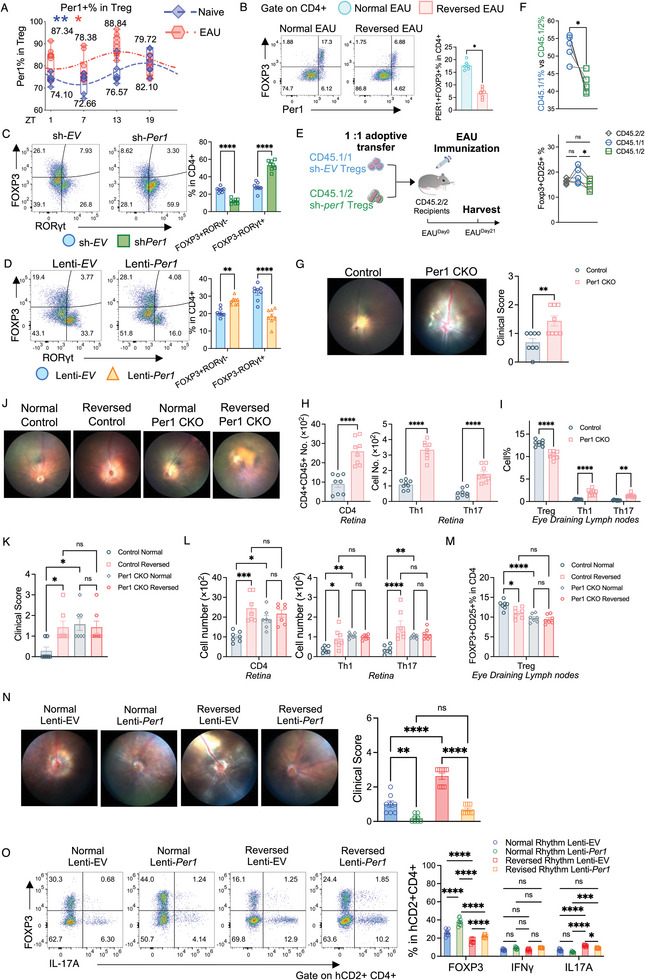
Circadian rhythm regulation of Treg function via Per1. A) The Plot display the percentages of Per1+ in Treg cells at different time points (ZT1, ZT7, ZT13, ZT19) and its fitted cosine curve in the eye‐draining lymph cells of Naïve and EAU mice. N = 7–10. Data was combined from two experiments. B) Representative FACS plots and the bar graph depict the percentage of Per1+FOXP3+ cells in Tregs of the eye‐draining lymph nodes from the Normal and the Reversed Groups. N = 6. Data was combined from two experiments. C) Treg (CD4+CD25+) cells were isolated from naïve C57BL/6J mice and transfected with sh‐Ctrl or sh‐*Per1* for 72 h in the presence of anti‐CD3/28 beads with IL‐6. Representative FACS plots and the bar graph presenting the FOXP3+RORγt‐, and FOXP3‐RORγt+ percentages in the sh‐*Per1* group. N = 8. Data was combined from two experiments. D) Treg cells were isolated from naïve C57BL/6J mice and transfected with Lenti‐*EV* or Lenti‐*Per1* in the presence of anti‐CD3/28 beads. Representative FACS plots and the bar graph presenting the FOXP3+RORγt‐, and FOXP3‐RORγt+ percentages. N = 8. Data was combined from two experiments. E, F) Treg cell adoptive transfer assay. E) Experimental scheme showing adoptive transfer of Treg cells with CD45.1/1+ sh‐*Ctrl* cells and CD45.1/2+ sh‐*Per1* cells to CD45.2/2+ EAU recipients. Treg cells were isolated from the spleens and lymph nodes of naïve CD45.1/1 and CD45.1/2 mice respectively, CD45.1/1 Tregs were treated with sh‐*Ctrl* and CD45.1/2 Tregs were cultured in the presence of sh‐*Per1* lentiviral‐transduction system. Later, CD45.1/1 and CD45.1/2 Tregs were collected and transferred to the CD45.2/2+ recipients in a ratio 1: 1. The CD45.2/2 recipients performed EAU induction on the same day. F) Frequency of CD45.1/1+ and CD45.1/2+ cells and FOXP3+CD25+ in (E) on the peak day of the EAU. N = 5. G–I) Per1^flox/flox^ Foxp3^YFP‐wt/wt^ (Control) and Per1^flox/flox^ Foxp3^YFP‐iCre/wt^ (Per1 CKO) mice were randomly divided into naïve and EAU groups respectively. Mice from the EAU group were immunized for EAU on Day 0 and sacrificed at ZT1 of Day 21. N = 8. Data was combined from two experiments. G) Representative fundus images from the control and Per1 CKO mice were taken on Day 20 after immunization. The bar graph showed the clinical scores. H) Bar graphs depicting the cell number of CD45+CD4+ cells, Th1 (CD4+ IFN‐γ+) cells, and Th17 (CD4+ IL‐17A+) cells from the retina of the Normal and the Reversed EAU mice on Day 21 after immunization. I) The bar graph depicting the percentage of Tregs (CD25+Foxp4+ in CD4), Th1 (IFN‐γ+ in CD4), and Th17 (IL‐17A+ in CD4) cells from the retina of the Normal and the Reversed EAU mice on Day 21 after immunization. J–M) Control and EAU mice were randomly divided into the Normal and Reversed groups respectively. All mice were immunized for EAU on Day 0 and sacrificed at ZT1 of Day 21. N = 7. Data was combined from two experiments. J, K) Representative fundus images were taken on Day 20 after immunization. The bar graph showed the clinical scores. L) Bar graphs depicting the cell number of CD45+CD4+ cells, Th1 (CD4+ IFN‐γ+) cells, and Th17 (CD4+ IL‐17A+) cells from the retina of indicated groups of EAU mice on Day 21 after immunization. M) Bar graphs depicting the percentage of Tregs (CD25+FOXP3+ in CD4) from the eye‐draining lymph nodes of the indicated groups on Day 21 after immunization. N, O) Tregs from the Normal Group and the Reversed Group were isolated and transfected with Lenti‐*EV* or Lenti‐*Per1*, respectively. After 24 h post‐transfection, cells were collected and injected into C57BL/6J mice which were immunized with hIRBP_1‐20_ on the same day. N = 8. Data were combined from two experiments. N) Representative fundus images displaying the effect of each group's Tregs in alleviating retinal inflammation. The bar graph shows the clinical scores in the indicated groups. O) Representative FACS plots and bar graphs depict the proportion of Treg cells, Th1, and Th17 cells in the hCD2+ cells from the indicated groups. A) Cosine similarity analysis and statistical significance were determined by Cosinor. Statistical significance was determined by Welch's *t*‐test in (B, G), upper box of (F), and left box of (H). Statistical significance was determined by two‐way ANOVA followed by Bonferroni test in (C,D,I–O), lower box of (F), and right box of (H). Data presented as means ± SEM, with *p < 0.05, **p < 0.01, ***p < 0.001, ****p < 0.0001.

Single‐cell RNA sequencing (scRNA‐seq) analysis showed that *Per1* has the highest expression among all other core clock genes, with a dramatic decrease in the Reversed Group in the Treg population (Figure S4A, Supporting Information). Statistically, *Per1* and *Bhlhe40* displayed significant differences in the Treg population in the eye‐draining lymph nodes (Figure S4B, Supporting Information). Validation of these core‐clock genes confirmed a significant decrease in *Per1*, *Bhlhe40*, and *Cry1* expression in eye‐draining lymph nodes (Figure S4C, Supporting Information). Notably, only PER1, not BHLHE40 or CRY1, exhibited significant differences at the protein level in eye‐draining lymph nodes (Figure S4D–F, Supporting Information). Flow cytometry analysis revealed that Per1+ Treg cells exhibited significant rhythmicity in eye‐draining lymph nodes of EAU mice (**Figure** **4**A). In the retina, Per‐1+ Treg cells exhibit rhythmic oscillations in both naïve and EAU mice. In addition, Per1+ CD4 cells, not other cell populations, exhibit rhythmic oscillations in EAU mice (Figure S4G, Supporting Information). However, the rhythmic oscillations were attenuated in the Reversed Group (Figure S4H, Supporting Information). Furthermore, flow cytometry plots showed the majority of FOXP3+ cells were PER1‐expressed cells (Figure 4B). In conclusion, Per1 is the most affected core clock gene in Treg populations. Its expression is significantly decreased in the Reversed Group. Rhythmicity in Per1+ Treg cells is observed in both naïve and EAU mice but attenuated in the Reversed Group. This underscores the impact of altered circadian rhythms on Treg function.

PER1, a core gene in the circadian rhythm, has previously been implicated in the cell cycle.^[^ ^32^ ^]^ To test if PER1 affects Treg cell stability, we conducted knockdown and overexpression experiments in vitro in the presence of IL‐6. We checked FOXP3 and RORγt expression, which are the functional transcription factors of Treg and Th17 respectively.^[^ ^33^, ^34^ ^]^ Following sh‐*Per1*, CD4+CD25+ T cells exhibited a significant decrease in the percentage of FOXP3+ RORγt‐ cells (Figure 4C). Conversely, in the Lenti‐*Per1* group, we observed a significantly higher level of FOXP3+ RORγt‐ cells and a lower level of FOXP3‐ RORγt+ cells in percentage compared to the Lenti‐*EV* group (Figure 4D). These data indicated that Per1 potentiates Treg stability.

To further corroborate that PER1 can regulate Treg cells in vivo, we isolated Treg cells from CD45.1/1 and CD45.1/2 mice and performed the knockout of *Per1* in the CD45.1/2 Tregs. After transfection, the same ratio (50% verse 50%) of CD45.1/1 (Per1‐sufficient) Treg and CD45.1/2 (Per1‐deficient) Treg were adoptively transferred together to CD45.2/2 EAU recipients at Day 0 (Figure 4E). At the peak disease, the transplanted cells with knock‐out of *Per1* (CD45.1/2+) exhibited decreased Treg cell percentage and FOXP3 expression (Figure 4F). Next, we generated Per1^flox/flox^ FOXP3^YFP‐iCre/wt^ (short for Per1 CKO) mice, which knockout Per1 in the Treg cells, to further prove the cell‐intrinsic role of Per1 in Tregs in vivo. The control and Per1 CKO mice were immunized for EAU on day 0. The fundus image was taken on Day 20 and mice were sacrificed on Day 21. The fundoscopy showed higher clinical scores in Per1 CKO mice (Figure 4G). Flow cytometer data showed more infiltrating CD45+ CD4+ T cells in the retina of Per1 CKO mice, specifically more Th1 and Th17 cells (Figure 4H), compared to the control mice. In eye‐draining lymph nodes, Per1 CKO mice exhibited lower percentages in the Tregs, and significantly higher percentages in both Th1 and Th17 cells (Figure 4I).

Next, to identify whether Per1 was required for Treg cells' circadian‐governed suppressive function, we established the EAU model in normal and reversed mice with or without Per1‐CKO. In the Per1‐CKO strain, both sub‐groups exhibited more severe retinal inflammation (Figure 4J,K). More CD45+ CD4+ T cells infiltrated the retina in the Per1‐CKO strain. However, subsequent circadian reversion also did not exacerbate this retinal infiltration (Figure 4L). Besides, Per1 was also required for the maintenance of Treg cell percentage in eye‐draining lymph nodes (Figure 4M). These data proved that Per1 was indispensable for rhythm‐dependent Treg cell proportion and function to limit inflammation response in autoimmune diseases.

Furthermore, we conducted a rescue experiment to verify whether PER1 is the key molecular mediator responsible for impaired Treg cell immunoregulatory function in circadian rhythm disruption. Tregs isolated from naïve mice under normal or reversed light exposure conditions were treated with either Lenti‐*EV* or Lenti‐*Per1*. Two days after transfection, cells were collected and transferred to EAU mice. After 14 days, fundoscopy revealed slight inflammation in the Normal Rhythm Tregs with Lenti‐EV, while severe inflammation with white exudations was observed in the retina of mice in the Reversed Rhythm Tregs with Lenti‐*EV*. Notably, there was minimal inflammatory evidence in the Reversed Rhythm Tregs with the Lenti‐*Per1* Group (Figure 4N). Flow cytometry analysis demonstrated that FOXP3 and IL‐17A expression patterns were consistent with the clinical score, with no differences in IFN‐γ expression (Figure 4O). These results collectively demonstrate that the cell‐intrinsic core‐clock gene *Per1* is pivotal for maintaining Treg cell stability and anti‐inflammatory function.

PER1 is known as a suppressor of BMAL1‐CLOCK complexes, and it has a complex network between different core circadian genes. Then, we wondered if the reversed lighting affects the Treg function only through PER1 or depends on other circadian components. We performed the double transfection experiments, knocking out each core clock gene with or without the presence of sg‐*Per1* (Figure S5A, Supporting Information). The data showed that in the sg‐*Bmal1* and *sg‐Clock* conditions, Treg stability was significantly decreased, and the additional sg‐*Per1* would not further decrease Treg's stability (Figure S5B, Supporting Information). This result indicated that *Bmal1* and *Clock* could affect Treg function as well. However, we did not detect the gene expression variation of *Bmal1* or *Clock* in our normal versus reversed lighting cycle mice (Figure S4A,B, Supporting Information). In summary, these data indicated that although *Bmal1* or *Clock* also can affect Treg stability, while in our Reversed lighting model and EAU mice, it may affect the circadian rhythm only starting from *Per1*.

In conclusion, our findings suggest that *Per1* plays a central role in regulating Treg stability and function. The lighting‐related Per1‐dependent Treg cells are essential in autoimmune inflammation.

### 2.5 Impaired Mitochondrial Function of Treg Cells due to Circadian Disruption via PER1

**Figure 5:**
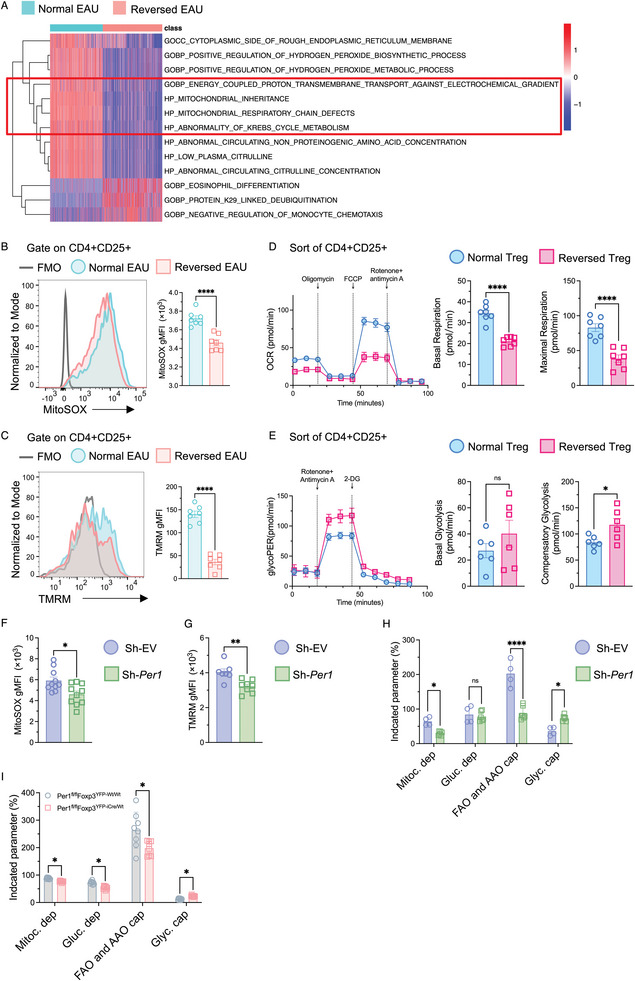
The impaired mitochondrial function of regulatory T cells due to circadian disruption via Per1. A) Heatmap depicting the pathways from Gene Set Variation Analysis (GSVA) with a LogFC threshold of >0.6 between the Tregs from the Normal EAU Group and the Reversed EAU Group. B, C) Representative FACS plots and bar graphs showing the geometric mean fluorescence intensity (gMFI) of MitoSOX and TMRM in the Treg cells from the Normal group and the Reversed group. N = 7. Data was combined from two experiments. D) OCR (oxygen consumption rate) assessment of the normal rhythm and the reversed rhythm Treg cells from naïve mice. Cells were sorted from eye‐draining lymph nodes before the OCR measurement. The OCR was measured following Oligomycin (2.5 µm), FCCP (2.5 µm), and Rotenone+ antimycin A (1 µm) treatment. Spare Respiration Capacity (OCR_max_–OCR_basal_) and ATP‐production Coupled Respiration (OCR_basal_–OCR_oligo_) were calculated and displayed in bar plots. N = 7. Data was combined from two experiments. E) Treg cells isolated from the normal rhythm of naïve mice were subjected to GlycoPER assay. Bar plots show basal glycolysis and compensatory glycolysis of normal rhythm Tregs and the reversed rhythm Tregs. N = 7. Data was combined from two experiments. F–H) Treg cells isolated from the normal rhythm of naïve mice were transfected with sh‐*Ctrl* and sh‐*Per1* for 72 h. Representative FACS plots and bar graphs showing the geometric MFI of MitoSOX (F) and TMRM (G) in the Treg cells from the sh‐*Ctrl* or sh‐*Per1* Group. N = 7–10. Data was combined from three independent experiments. H) SCENITH assay was performed for metabolic assessment of Treg cells with sh‐*Ctrl* and sh‐*Per1*. Oligomycin (1 µm), 2‐DG (100 mm), or Oligomycin (1 µm) + 2‐DG (100 mm) were added for 45 min in the respective wells. Puromycin was added to co‐cultures for the last 30 min, and then Treg cells were stained with anti‐puromycin. The MFI of puromycin was collected and calculated. N = 4–6. Data was combined from two independent experiments. I) Treg cells were isolated from the eye‐draining lymph nodes of control and PER1 CKO mice. SCENITH assay was performed for metabolic assessment. N = 8. Data was combined from two independent experiments. B–G) Statistical significance was determined by Welch's *t*‐test. H, I) Statistical significance was determined by two‐way ANOVA followed by the Bonferroni test. Data presented as mean ± SEM, with *p < 0.05, **p < 0.01, ***p < 0.001, ****p < 0.0001.

In our Gene Set Variation Analysis (GSVA) with a LogFC threshold of >0.6, we identified that differentially expressed genes in EAU mice pointed to impaired mitochondrial function in Tregs from the Reversed Group (**Figure** **5**A). To delve into this phenomenon, we conducted a series of experiments. The geometric mean fluorescence intensity (gMFI) and percentage of the mitochondrial superoxide indicator (MitoSOX) were significantly decreased in Tregs from EAU mice with reversed circadian rhythm (Figure 5B, Figure S6A, Supporting Information). Simultaneously, staining with tetramethylrhodamine methylester (TMRM), which assesses mitochondrial membrane potential (ΔΨm),^[^ ^35^ ^]^ revealed a reduction in Tregs from EAU mice in the Reversed Rhythm Group (Figure 5C, Figure S6B, Supporting Information).

T cells can generate ATP through oxidative phosphorylation (OXPHOS) and glycolysis pathways.^[^ ^36^ ^]^ Tregs are known to predominantly generate ATP through OXPHOS, while Th1/Th17 cells primarily rely on glycolysis.^[^ ^37^ ^]^ Modulating the metabolic pathways of Treg cells, specifically by reducing oxidative phosphorylation while increasing glycolysis, can induce Treg instability.^[^ ^38^ ^]^ To investigate if OXPHOS is affected in the Tregs of the Reversed Group, we conducted a Mito Stress Test and metabolic flux analysis. The results revealed a significant decrease in the maximal oxygen consumption rate (OCR), spare respiration capacity (OCR_Max_ – OCR_Basal_), and ATP‐production coupled respiration (OCR_Basal_ – OCR_Oligomycin_) in the Tregs from the Reversed Group compared to the Normal Group (Figure 5D, Figure S6C, Supporting Information). In contrast, the glycolytic proton efflux rate (glycoPER), which measures glycolysis, showed a significant increase in compensatory glycolysis in the Reversed Tregs, with no significant difference in basal glycolysis between the two groups (Figure 5E).

We further investigated whether PER1 was responsible for rhythm‐disruption‐induced mitochondrial dysfunction. In Per1‐deficient Tregs, we observed a significant decrease in the geometric mean fluorescence intensity (gMFI) of MitoSOX and TMRM (Figure 5F,G). To explore this in‐depth, we utilized the SCENITH assay^[^ ^39^ ^]^ to measure puromycin MFI in Tregs treated with sh‐*EV* or sh‐*Per1* lentiviral transduction system and Tregs isolated from the Per1^fl/fl^ Foxp3^YFP‐iWt/Wt^ versus Per1^fl/fl^ Foxp3^YFP‐iCre/Wt^ (PER1 CKO) mouse. Our results demonstrated that in the sh‐*Per1* group, there was a significant reduction in OXPHOS (mitochondrial dependence, Mitoc. dep.) compared to the sh‐*EV* group, along with a notable decrease in fatty acid oxidation and amino acid oxidation capacity (FAO and AAO cap.), albeit no differences in glucose oxidation (Gluc. dep.). Additionally, there was a significant increase in glycolytic capacity (Glyc. cap), representing the maximum capacity of glycolysis when mitochondrial OXPHOS is inhibited (Figure 5H). Similarly, Tregs from Per1‐CKO mice showed a significant decrease in Mitochondria dependence, Glucose dependence, and FAO and AAO capacity compared to the Tregs from the control mice, along with an increase in glycolytic capacity (Figure 5I). These findings collectively underscored the detrimental impact of circadian rhythm disruption on Treg mitochondrial function, with PER1 playing a pivotal role in this process.

### 2.6 PER1 Modulates Treg Stability Through COX7C

In our scRNA‐seq, we observed a series of mitochondrial‐related genes with significant decreases in Tregs from the Reversed Rhythm EAU mice (Figure S4A, Supporting Information). We first validated the expression of these mitochondrial‐related genes using RT‐qPCR. Among these genes, *Cox7c* exhibited significant decrease in the Reversed EAU mice (Figure S7A, Supporting Information). Cytochrome c oxidase (COX), the terminal component of the mitochondrial respiratory chain, plays a pivotal role in catalyzing the electron transfer from reduced cytochrome c to oxygen, where COX7C serves as one such subunit and is expressed in all tissues.^[^ ^40^ ^]^

**Figure 6:**
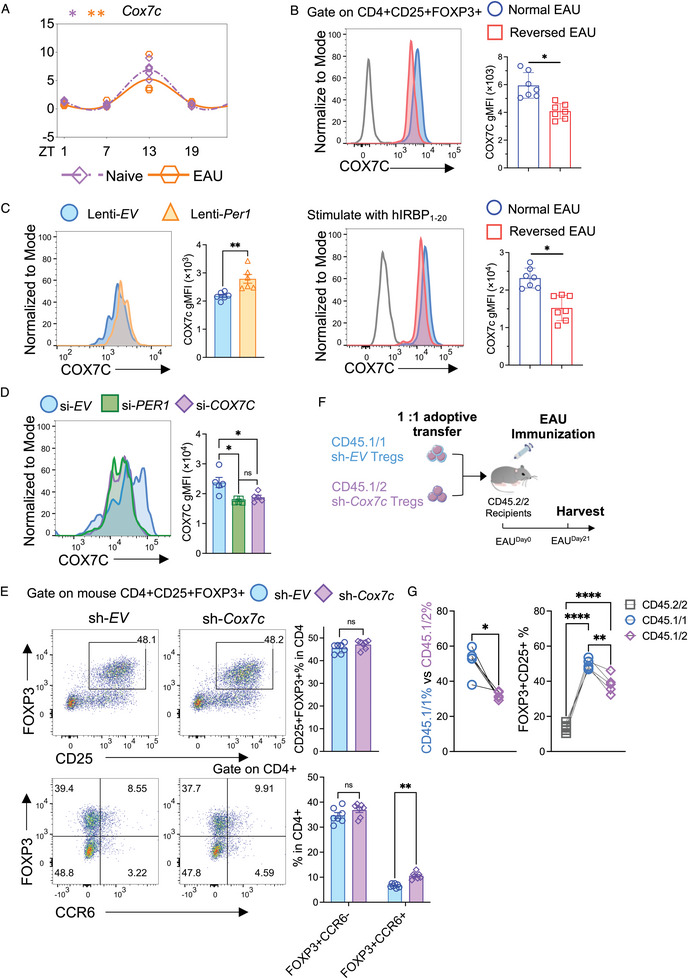
PER1 modulates Treg stability through COX7C. A) The plot showing the expressions of *Cox7c* at different ZT time points and its fitted cosine curve. N = 6–8. Data was combined from two experiments. B) Representative FACS plots and bar graphs presenting geometric gMFI of COX7C in vivo and in vitro from Treg cells in the Normal group and the Reversed group. N = 7. Data was combined from two experiments. C) Representative FACS histogram and the bar graph showing Cox7c levels in Treg cells transfected with Lenti‐*EV* or Lenti‐*Per1*. N = 6. Data was combined from two experiments. D) Representative FACS histogram and the bar graph displaying the geometric MFI of COX7C in the isolated human Treg cells from PBMC treated with non‐target siRNA (si‐*Ctrl*), si‐*PER1*, or si‐*COX7C* for 72 h. N = 5. Data was combined from two experiments. E) Sh‐*Ctrl* or sh‐*Cox7c* was added to isolated mouse Treg cells for 72 h. Representative FACS histogram and bar graphs displaying the percentage or cell number of the CD25+FOXP3+, FOXP3+CCR6‐, FOXP3+CCR6+ population with sh‐*Cox7c*. N = 7. Data was combined from two experiments. F) Experimental scheme showing adoptive transfer of Treg cells with CD45.1/1+ sh‐*Ctrl* cells and CD45.1/2+ sh‐*Cox7c* cells to CD45.2/2+ EAU recipients. G) Frequency of CD45.1/1+ and CD45.1/2+ cells and FOXP3+ CD25+ in (F). N = 5. A) Cosine similarity analysis and statistical significance were determined by Cosinor. B,C) Statistical significance was determined by Welch's *t*‐test. D,E) Statistical significance was determined by two‐way ANOVA followed by the Bonferroni test. G) Statistical significance was determined by Paired *t*‐test. Data presented as mean ± SEM, with *p < 0.05, **p < 0.01, ***p < 0.001, ****p < 0.0001.

Our diurnal variation experiments revealed the rhythmic expression of Cox7c (**Figure** **6**A). Furthermore, at the protein level, flow cytometry measurements of the geometric MFI (gMFI) of COX7C in Tregs from both in vivo and in vitro settings corroborated the reduction in COX7C expression in circadian‐disrupted Tregs (Figure 6B). Additionally, a majority of FOXP3+ cells expressed Cox7c, and the FOXP3+COX7C+ population was reduced in the Reversed Group (Figure S7B, Supporting Information).

In the *Per1*‐overexpression experiments, we observed a significant increase in COX7C gMFI in the Lenti‐*Per1* group (Figure 6C). To explore whether COX7C expression is regulated by PER1, Treg (CD4+CD25+) cells were sorted and treated with si‐*Control*, si‐*Per1*, or si‐*Cox7c*. COX7C expression was significantly decreased in the Per1 knockdown condition, with no significant differences observed in the si‐*Cox7c* group, suggesting the maintenance of COX7C expression was required with PER1 (Figure 6D). Si‐*Cox7c* treatment on Treg cells resulted in an increase in the FOXP3+ CCR6+ population compared to the control group among mouse Treg (CD4+CD25+) cells (Figure 6E).

In vivo, we conducted the adoptive transfer experiment to further confirm the role of COX7C in Treg cells. As the gating strategy is shown in Figure S7C (Supporting Information), control (CD45.1/1) and *Cox7c‐*deficient Treg cells (CD45.1/2) to CD45.2/2 EAU recipients (Figure 6F). At the peak disease, the transplanted cells with knock‐out of *Cox7c* exhibited impaired Treg cell percentage and FOXP3 expression (Figure 6G). These findings revealed that circadian disruption‐induced PER1 decrease may contribute to impaired mitochondrial function through its influence on COX7C, ultimately impacting Treg stability.

### 2.7 PER1/COX7C Maintains Human Treg Stability

**Figure 7:**
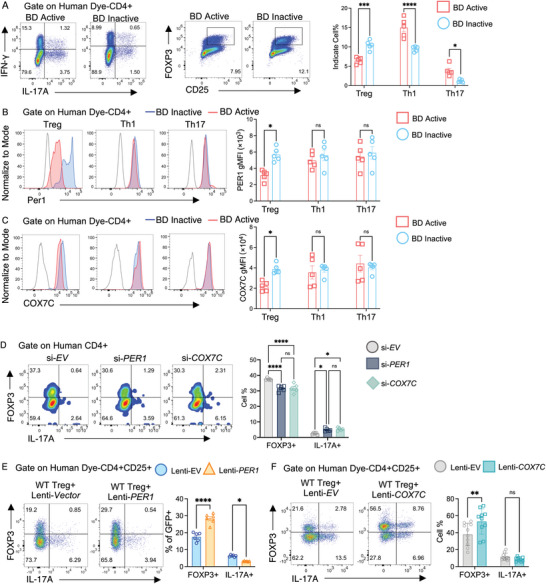
PER1/COX7C modulates human Treg stability. A) Representative FACS plots and the bar plot showed Treg, Th1, and Th17 percentages in CD4+ T cells from healthy controls, BD active, and BD inactive patients. N = 5. Data were combined from two experiments. B,C) Representative histograms and the bar plot showed PER1 and COX7C geometric mean fluoresce index (MFI) in Tregs, Th1, and Th17 Cells from healthy controls, BD active, and BD inactive patients. N = 5. Data were combined from two experiments. D) Representative FACS histogram and the bar graph displaying the percentage of FOXP3+ and IL‐17A+ in the isolated human Treg cells treated with non‐target siRNA (si‐*CTRL*), si‐*PER1*, or si‐*COX7C* for 72 h. N = 5. Data were combined from two experiments. E) Human CD25+CD4+ cells were isolated from the PBMC of healthy donors and transfected with Lenti‐*EV‐GFP* or Lenti‐*PER1‐GFP* and cultured for 72 h in the presence of hIL‐2. Representative FACS plots and bar graphs show the cell percentage and cell number of Treg and Th17 cells in the GFP+ population. N = 6. Data were combined from two experiments. F) Human CD25+CD4+ cells were isolated from the PBMC of healthy donors and transfected with Lenti‐*EV‐mCherry* or Lenti‐*COX7C‐mCherry* and cultured for 72 h in the presence of hIL‐2. Representative FACS plots and the bar graph show the cell percentage of Treg and Th17 cells in the mCherry+ population. N = 10. Data were combined from three experiments. A–F) Statistical significance was determined by two‐way ANOVA followed by the Bonferroni test. Data presented as mean ± SEM, with *p < 0.05, **p < 0.01, ***p < 0.001, ****p < 0.0001.

To further confirm whether Per1/Cox7c modulated Treg stability in the patients, we collected peripheral blood monocular cells (PBMC) from Bechet's disease (BD) patients with or without active disease activity. As expected, active BD patients showed a significant decrease in the Treg population and an increase in Th1 and Th17 proportion compared to inactive patients (**Figure** **7**A). Flow cytometry analysis revealed a significantly lower gMFI of PER1 and COX7C in active BD patients compared to other groups in the Treg population, but not in Th1 or Th17 cells (Figure 7B,C).

To further confirm the role of PER1 and COX7C in human Tregs, we also performed a Treg stability experiment in the knockdown and overexpression conditions in vitro. Si‐*Cox7c* treatment on human CD25+ cells led to decreased FOXP3 expression and increased IL‐17A production (Figure 7D). Similar to the data in mice, over‐expression of both PER1 and COX7C gene in human Treg cells increased the FOXP3+ population and reduced IL‐17A production (Figure 7E,F). Thus, these results suggested a positive correlation between COX7C^high^ Tregs and uveitis severity.

## 3 Discussion

Growing evidence indicates that disturbance of circadian rhythm aggravates autoimmune diseases.^[^ ^38^ ^]^ In this study, we found that Treg cells oscillate rhythmically in EAU mice. In the reversed lighting system, we found that the EAU was more severe due to the decreased Treg population and function. Mechanically, the circadian clock disruption impaired Treg cell stability and function through the core‐clock gene *Per1*‐associated *Cox7c*‐dependent mitochondria metabolic process. Briefly, this study highlights that the circadian clock can directly control the immunosuppressing function of Treg through the core‐clock gene *Per1*‐dependent mitochondrial metabolism in autoimmune disease.

Typically, researchers use *Bmal1* or *Clock* deficient mice for circadian studies on autoimmune diseases.^[^ ^11^, ^13^, ^25^, ^41^ ^]^ However, this approach may overlook the potential involvement of other core‐clock genes. Moreover, this research method presents challenges in replicating clinical realities. The light‐dark cycle is crucial for circadian rhythms, and recent studies have underscored the repercussions of altering this cycle.^[^ ^42^ ^]^ Nowadays, manipulation of the artificial light‐dark cycle stands as a prevalent method in circadian‐related research.^[^ ^20^, ^43^, ^44^, ^45^ ^]^ In this study, we placed EAU mice into the normal or reversed light‐dark cycle, which can more realistically simulate how light rhythm disruption (similar to jet lag) would affect uveitis disease. A notable decrease in Treg cell percentages and numbers was observed in the reversed group. Furthermore, in vivo and in vitro function experimental results revealed that circadian disruption led to impairments in Treg percentage, stability, and immunosuppressing function, thus heightening uveitis severity regarding clinical scores and pathological levels. Our research demonstrates that the manipulation of the light‐dark cycle exacerbates autoimmune uveitis, aligning with the clinical observation that disruptions in circadian rhythms contribute to disease aggravation and recurrence.

In autoimmune diseases, such as inflammatory bowel disease (IBD), SLE, and RA, T cells are considered the major effector cells.^[^ ^46^, ^47^ ^]^ Previously, literature has described the dynamic regulation of clock activity in T cells.^[^ ^21^, ^22^, ^48^ ^]^ However, it seems that any oscillations of clock genes in naïve CD4+ T cells are weak. It has been reported that the rhythmicity of naïve T cells could be impaired by T cell‐specific ablation of *Bmal1*.^[^ ^49^ ^]^ Nevertheless, another report claimed that the cell‐intrinsic clock is dispensable in the naïve CD4 T cells.^[^ ^23^ ^]^ Despite the lack of conclusive evidence of a functional clock within naïve CD4 T cells, there is clear evidence that the cell‐intrinsic clocks of autoreactive T cells regulate autoimmune diseases.^[^ ^22^, ^25^, ^49^ ^]^ In Teffs, molecular clock gene Rev‐erb alpha in Th17 cells has been reported to control cell differentiation, development, and autoimmunity,^[^ ^13^, ^24^ ^]^ yet whether there is a functional cell‐intrinsic circadian clock in Tregs is controversial. A recent study claimed that naïve Treg cells do not possess functional clock machinery.^[^ ^25^ ^]^ In the current study, we noticed rhythmic oscillations in the CD3 and CD4 of EAU mice. Importantly, when we zoomed into the subsets of CD4+ T cells, we observed diurnal expressions of Tregs in both naïve and EAU mice, but not in the Teffs. Our findings indicate that circadian rhythm has a greater impact on Tregs in EAU mice.

Next, we moved on to which gene is important for circadian rhythm‐controlled Treg cells. It is essential to note that many circadian studies have primarily focused on the specific deletion of the gene *Bmal1* in T cells. However, we did not observe a significant difference in the expression of *Bmal1* in single‐cell RNA‐seq and RT‐qPCR validation in the eye‐draining lymph nodes of EAU mice from the Normal and the Reversed Groups (Figure S4A,B, Supporting Information). However, when we knocked down each circadian gene in vitro, we found that sg*‐Bmal1* and/or sg*‐Clock* alone could decrease Treg stability. These gave us a hint that *Bmal1* and *Clock* are also important for Treg stability but are not involved in the reversed lighting disruption model here. Interestingly, our data showed increased stability of Tregs in the presence of sg‐*Cry1* (Figure S5A, Supporting Information), which indicated that Cry1 may have a negative role in Treg stability.

However, there is no significant expression difference of Cry1 at the protein level in the Treg cells from the Normal and the Reversed Groups (Figure S4F, Supporting Information), thus we did not put more emphasis on it, which remains further investigation. These findings suggest that while Cry1 may influence Treg stability, additional factors may also play a crucial role, warranting more comprehensive studies to elucidate its exact function.

Among all core circadian genes, *Per1* is the only core‐clock gene that exhibits rhythmic oscillations in the eye‐draining lymph nodes of both naïve and EAU mice. Additionally, the single cell‐RNA‐seq data and expression validation results showed that only PER1 is significantly decreased in the Tregs from the Reversed Group. Furthermore, flow cytometry data showed the PER1 expression in the Tregs of EAU mice expressed rhythmically, which is in line with the previous finding that light exposure causes increases in PER1 mRNA.^[^ ^50^ ^]^ In total, our data indicated that PER1 in Treg cells plays a vital role in the circadian‐dependent EAU.

PER1, one of the key characteristic circadian oscillators,^[^ ^51^ ^]^ has been recognized previously for its role in the cell cycle and its anti‐tumor effect.^[^ ^52^ ^]^ Biopsy analysis of inflamed tissue of IBD patients showed that *Per1* levels were significantly decreased compared to the healthy control,^[^ ^53^ ^]^ but PER1 was up‐regulated in peripheral blood of MS patients.^[^ ^54^ ^]^ To date, there is limited literature addressing the role of PER1 in CD4+ T cells. A recent report showed stress hormones induce the expression of PER1 to inhibit Th1 cytokine expression via mTORC1 in naïve CD4 T cells.^[^ ^55^ ^]^ The role of PER1 in Tregs has not been discovered. Here, by silencing *Per1* in Treg cells, we observed a significant impairment in stability and suppressing function in vitro. Using Per1 CKO mice, we can confirm the cell‐intrinsic role of Per1 in Treg and its role in EAU. Further, the rescue experiments proved that by overexpressing PER1 in the Tregs from the reversed group, they regained their ability to suppress autoimmune inflammation. Our results demonstrate that PER1 is essential for Treg stability and function, and circadian rhythm could control Treg function through PER1 directly.

Recent research underscores the essential role of cell metabolism in shaping the fate and functional states of T cells while highlighting diurnal fluctuations in mitochondrial energy metabolism and oxidative pathways.^[^ ^56^ ^]^ Conventionally, Teffs rely on aerobic glycolysis, while Tregs predominantly engage in oxidative phosphorylation.^[^ ^57^ ^]^ PER1 has emerged as a central regulator of cellular metabolism lately. *Per1* has shown its impact on the rhythmic oscillations of mitochondrial rate‐limiting enzymes,^[^ ^58^ ^]^ the preservation of mitochondrial function,^[^ ^59^ ^]^ and the suppression of glycolysis.^[^ ^60^ ^]^ Consistent with other cells, we uncovered that *Per1*‐deficient Tregs exhibit reduced mitochondrial function and oxidative phosphorylation, which correlates with the decreased FOXP3+ proportion, in a *Cox7c*‐dependent manner. Thus, our findings point out that the PER1‐dependent Treg cell stability and function were determined by the mitochondrial metabolism.

COX7C is a subunit within mitochondrial complex IV, the terminal enzyme situated in the mitochondrial membrane that plays a crucial role in the respiratory electron transport chain.^[^ ^61^ ^]^ This complex consists of 14 protein subunits and serves as the regulatory hub for mitochondrial oxidative phosphorylation.^[^ ^62^ ^]^ COX7C is categorized as a long‐lived mitochondrial protein and plays a critical role in maintaining the mitochondrial proteome and ensuring robust mitochondrial function.^[^ ^63^ ^]^ Notably, in Type 2 Diabetes, COX7C has been associated with oxidative stress levels and is believed to contribute to the risk of disease development.^[^ ^64^ ^]^ However, the regulatory mechanisms governing COX7C expression remain largely unknown. Our mouse and human data collectively demonstrate that COX7C expression was determined by PER1 expression level (Figures 6D and 7C). These results indicate a positive correlation between COX7C^high^ Tregs and uveitis prognosis. Taken together, our findings underscore the significance of Per1‐dependent Cox7c in Treg function and its implications in autoimmune inflammation.

Our data also revealed that CD8+T cells expressed significant variations in the percentages of CD8+ cells in naive mice (Figure S1B, Supporting Information). Furthermore, the core clock gene expression indicated that there were several core clock proteins, including CLOCK, CRY2, NR1D1, and NR1D2 expressed rhythmically in CD8+ T cells and other immune cell types in the eye‐draining lymph nodes of naïve and EAU mice (Figure S8 and Table S1, Supporting Information). However, the phenotype needs further validation, and the possible mechanism needs further investigation. As uveitis is more related to CD4+ T cells and there were no significant variations in the percentages of other immune cell types in EAU mice, we were not going to further investigate in this paper.

## 4 Conclusion

In summary, our research provides a potential explanation for the aggravation of autoimmune uveitis due to disrupted circadian rhythms. Our study introduces a novel hypothesis suggesting that alignment of activity with the light‐dark clocks is responsible for maintaining the expression of the core‐clock gene *Per1*, thereby ensuring cellular stability, immunosuppressive function, and oxidative phosphorylation in Treg cells. Consequently, our findings strengthen the link between Treg cell function, circadian rhythms, and cellular metabolism, proposing that interventions aimed at circadian and metabolic pathways hold promise as strategies to modulate Treg cell functionality in autoimmune diseases.

## 5 Experimental Section

### Ethical Statement

CD45.1/1, CD45.1/2, and CD45.2/2 C57BL/6J (shortened to WT) from 6 to 8 weeks old, were purchased from Guangdong Gempharmatech (Gempharmatech Co., Ltd, Guangzhou, China). FOXP3tm4(YFP/cre) Ayr (No. 016959) mice (shortened to FOXP3 ^YFPicre)^ were purchased from the Jackson Laboratory. Per1‐Cas9‐KO (shortened to Per1‐CKO) was created by Nanjing Gempharmatech (GemPharmatech Co., Ltd, Nanjing, China). FOXP3YFPicre mice crossed with Per1‐CKO mice in Nanjing Gempharmatech. All mice are cared for experiments at the Zhongshan Ophthalmic Center of Sun Yat‐sen University. All animal experiments were approved by the Institutional Animal Care and Use Committee of Zhongshan Ophthalmic Center of Sun Yat‐sen University and were compliant with the Association for Research in Vision and Ophthalmology (ARVO) Statement for the Use of Animals in Ophthalmic and Vision Research (Approval No. O2021074).

Five active BD patients and five inactive BD patients were enrolled in this study at the Zhongshan Ophthalmic Center. The age of subjects ranged between 18 and 60 years (Table S4, Supporting Information). All patients were diagnosed based on the disease manifestations, the results of standard OCT, and fluorescein angiography according to the Revised Diagnostic Criteria. For active BD, it means there were floating cells in the anterior chamber or vitreous and/ or any manifestation of retinal vasculitis. If there was no cell in the anterior chamber or vitreous and no retinal vasculitis, the patient would be considered inactive BD. All patients collected blood samples in the morning ≈8–9 a.m.

The study received approval from the Human Research Ethics Committee of Zhongshan Ophthalmic Center of Sun Yat‐sen University (No. ID: 2020KYPJ104).

### Light‐Induced Circadian Rhythm Disrupting Model

Mice were randomly divided into two groups. The first group adhered to a conventional 12–12 light‐dark cycle (lights on at 7 a.m., lights off at 7 p.m., referred to as the “Normal Group”). The second group of mice followed a reversed 12–12 light‐dark cycle (lights on at 7 p.m., lights off at 7 a.m., referred to as the “Reversed Group”).

### EAU Immunization

Human interphotoreceptor retinoid‐binding protein 1–20 (hIRBP_1‐20_, 2 mg mL^−1^, GPTHLFQPSLVLDMAKVLLD, GL Biochem, Shanghai, China) was emulsified with complete Freund's adjuvant (CFA, Difco, MD, USA) containing *Mycobacterium tuberculosis* strain H37Ra extract (5 mg mL^−1^; Difco, MD, USA) in a 1:1 volume ratio. Each mouse was subcutaneously injected with 200 µL emulsion at the two different sites of the lower flanks and on the back for immunization. Pertussis toxin (5 ng per mouse; ListBiological Laboratories, CA, USA) was administered intraperitoneally on day 0 and day 2 post‐immunization. Mice were monitored by Micron IV Retinal Imaging Microscope (PHOENIX) on day 21 for clinical signs of EAU. The scores were graded as described before.^[^ ^65^ ^]^ At the end of the experiment, the mice were sacrificed at ZT1 or indicated ZT on day 22, and eyeballs and eye‐draining lymph nodes and spleens were collected. For H&E staining, eyeballs were isolated and underwent fixation with 4% paraformaldehyde overnight, and then dehydrated, embedded, and sectioned into 4 µm for further staining. The degree of retinal inflammation was microscopically measured and quantitatively analyzed as described before.^[^ ^65^ ^]^ For retina flow cytometer staining, retinas were isolated under the microscope.

### Flow Cytometry Analysis

Tissues were homogenized in 2% FBS in RPMI and filtered using 40 µm strainers. Cells were washed and stained with Zombie NIR Fixable Viability Kit (CAT #423106, BioLegend, San Diego, CA, USA). Cells were stained following the manufacturer's protocol.

For intracellular staining, Brefeldin A (BD GolgiPlug; BD Biosciences, Franklin Lake, NJ, USA) 1 µg mL^−1^, PMA (Sigma–Aldrich, St. Louis, MO, USA) 50 ng mL^−1^, and ionomycin (Sigma–Aldrich, St. Louis, MO, USA) 200 ng mL^−1^ was added for 6 h. Cells were stained following the manufacturer's protocol. Staining was performed as previously described, using the FOXP3 Staining Kit (Catalog #88‐8111‐40, eBioscience, Thermo Fisher Scientific, Waltham, MA, USA). Cells were collected for flow cytometry. All samples were acquired with BD LSR Fortessa Flow Cytometer (BD Biosciences, Franklin Lake, NJ, USA) and analyzed with FlowJo software (v10.4).

### Treg Cell Isolation

The Tregs were sorted using the mouse CD4+CD25+ Regulatory T‐cell Isolation Kit (STEMCELL Technologies, Cambridge, MA, USA). Cells were cultured in 10% FBS 1640 with anti‐CD3/CD28 beads (5 × 10^4^ per well, Invitrogen, Thermo Fisher Scientific, Waltham, MA, USA).

### Treg Cell Proliferation Assay

The CD4+CD25+ cells were isolated from the eye‐draining lymph nodes of naïve mice in the Normal and the Reversed Groups and were collected and labeled with Tag‐It Violet Proliferation Cell Tracking Dye (CAT#425101,1 µmol L^−1^, Biolegend, San Diego, CA, USA) in a 37 °C water bath for 10 min. The labeled cells were cultured in 96‐well plates at 2 × 10^5^ cells per well density with anti‐CD3/CD28 beads. Cells were cultured for 96 h and collected for flow cytometry analysis.

### Treg Suppression Assays

T cells isolated from the spleen and lymph nodes of WT mice were enriched using a commercial T‐cell Isolation Kit (Catalog #11413D, Invitrogen, Thermo Fisher Scientific, Waltham, MA, USA), then labeled with Violet Proliferation Cell Tracking Dye (CAT#425101,1 µmol L^−1^, BioLegend, San Diego, CA, USA). The CD4+CD25+ cells were isolated from the eye‐draining lymph nodes of naïve mice in the Normal and the Reversed Group. The labeled T cells (2 × 10^4^ cells) and Treg cells in various concentrations (1:1 to 1:16 Treg: T cell ratios) were then activated with anti‐CD3/CD28 beads for 72 h.

### Treg Stability Assays

The isolated Tregs cells were incubated with anti‐CD3/CD28 beads and incubated with IL‐2 (10 ng mL^−1^) with or without IL‐6 (50 ng mL^−1^) (Both Biolegend, San Diego, CA, USA) in 96‐well plates above 3 × 10^5^ cells per well for 72 h.

### Plasmids

Both human and murine full‐encoding sequences of proteins including PER1 were constructed and cloned into Flag‐tagged pCDH expression plasmids for the lentiviral over‐expression system. For the gene silencing of murine PER1 and COX7C, corresponding shRNA vectors were constructed using pLKO.1 backbone.

To trace the Per1‐overexpressed cells and EV control cells in vivo, an hCD2‐coexpressed vector cloned with or without Per1 coding sequence was applied for Per1‐overexpression and empty vector respectively. The constructed plasmids were all verified by sequencing. Then, the Per1‐hCD2 vector and the EV‐hCD2 vector were packaged into lentivirus and transfected to the sorted Treg cells respectively before adoptive transfer. Two weeks after the transfering, the lymphoid organs were harvested, and the transferred cells were traced by gating on CD4+ hCD2+ subset.

All the sequences of plasmids and shRNA are in Table S2 (Supporting Information).

### Lentivirus Transduction

HEK293T cells were cultured to 70–95% density in a 10‐cm dish with DMEM medium containing 10% FBS. For the lentivirus package, 2.5 µg pMD2.G, 7.5 µg psPAX2, and 10 µg expression vectors were co‐transfected to HEK293T cells with Trans‐EXP Liposomal Transfection Reagent (TranSheep Bio Co. Ltd). After 8 h of transfection, the cells were replaced with fresh medium, and the medium containing lentivirus was collected at 24 and 48 h. Finally, the lentivirus was concentrated by adding the medium with Lenti‐Pac Lentivirus Concentration solution (GeneCopoeia) and was resuspended using Opti‐MEM medium (Gibco). The T cells cultured in 1640 medium were mixed with the lentivirus solution at 1: 1 ratio in 96‐well plates pre‐coated with Retronectin (Takara). After transduction, the cells were washed three times and were kept in culture for further experiments.

### T cell Transfection

Human or murine T cells were transfected with the corresponding lentivirus. Briefly, 1 × 10^6^ per well cells were resuspended in 100 µL medium containing lentivirus at MOI of 200. Then the mixture was supplemented with 8 µg mL^−1^ polybrene (HB‐PB‐500, HANBIO) and placed in 96‐well plates pre‐coated with Retronectin (1 mg mL^−1^, Takara, T100A). The cells were cultured for 48 hour. After transfection, the cells were washed with PBS and cultured in fresh RPMI 1640 medium containing 0.5 × 10^5^ anti‐CD3/CD28 beads/well for further functional assay.

### ScRNA‐Sequencing

The cells derived from eye‐draining lymph nodes (Four mice in each Group) were washed with PBS three times and single‐cell suspensions were obtained after through a 40 µm cell strainer (Falcon). The cells were assessed on number and viability and were then applied to the generation of barcoded ScRNA‐seq libraries according to the manufacturer's instruction (10×Genomics). Next, the complementary DNA (cDNA) was generated from the released barcoded RNA via reverse transcription and was imported to Illumina sequencing libraries and sequenced on a NovaSeq6000 platform (10×Genomics). The ScRNA‐seq data was checked by FastQC software and converted to a fastqs file through cell ranger (v5.1.0; 10×Genomics).

### ScRNA‐Seq Raw Data Processing and Cell‐Type Annotation

The scRNA‐seq raw data were counted and aggregated in CellRanger (https://support.10xgenomics.com/single‐cell‐gene‐expression/software/pipelines/latest/using/count for count, and https://support.10xgenomics.com/single‐cell‐gene‐expression/software/pipelines/latest/using/count for aggregation) to generate gene expression matrices.

The data was analyzed in R software (v.4.0.3) with R package Seurat (v4.0.1). After quality control, normalization, and variable gene identification, the data were subjected to dimensional reduction using the RunTSNE function. The distinguishable genes of each cell cluster were calculated by the Seurat FindAllMarker function. Each cluster was annotated based on the markers of major cell types.

### DEG Identification and Gene Set Variation Analysis

The R package limma (v3.44.3) was used to identify differentially expressed genes (DEGs). For functional activity estimation, R package GSVA (v1.36.3) was employed to calculate the scoring activity in each cell according to the molecular signature database (https://www.gsea‐msigdb.org/gsea/msigdb/genesets.jsp). The genes with logarithmic fold change over 0.25 and *p*‐value <0.05 were considered significant.

### Statistical Analysis

GraphPad Prism 9.1.1 was used for statistical analyses. Welch's *t*‐test and Mann–Whitney U test were used as indicated in the figure and/or legends. N values and replicate experiments are all located in figure legends. *p < 0.05, **p < 0.01, ***p < 0.001.

## Conflict of Interest

The authors declare no conflict of interest.

## Author Contributions

W.Z., G.C., Z.X., and M.W. contributed equally to this work and should be considered co‐first authors. W.Z., G.C., Z.X., and M.W. designed the study, analyzed the data, and wrote the manuscript. Y.S., Z.L., and X.L. assisted in the experiment methodology. Z.L., and H.H. provided guidance with the individual experiments and supervised the study. X.C., L.L., and D.L. were responsible for the conception and design, revision of the manuscript, and approval of the final manuscript. All authors read and approved the final manuscript. And approved the version submitted for publication.

## Supporting information



Supporting Information

## Data Availability

The data that support the findings of this study are available from the corresponding author upon reasonable request.
