# Supplementary material for: Circadian Rhythm Disruption Exacerbates Autoimmune Uveitis: The Essential Role of PER1 in Treg Cell Metabolic Support for Stability and Function
Source: Adv Sci (Weinh). 2025 Jan 17;12(10):2400004. doi: 10.1002/advs.202400004 (PMC11904989; doi:10.1002/advs.202400004)
Supplement: Supplementary file 1 — Supporting Information [file ADVS-12-2400004-s001.docx]

Supporting Information

**Circadian Rhythm Disruption Exacerbates Autoimmune Uveitis: The Essential Role of PER1 in Treg Cell Metabolic Support for Stability and Function**

*Wenjie Zhu#, Guanyu Chen#, Zhiqiang Xiao#, Minzhen Wang#, Yuxun Shi, Zhuang Li, Xiaohui Luo, Zuoyi Li, Haixiang Huang, Xiaoqing Chen*, Lingyi Liang*, Dan Liang*.*

**
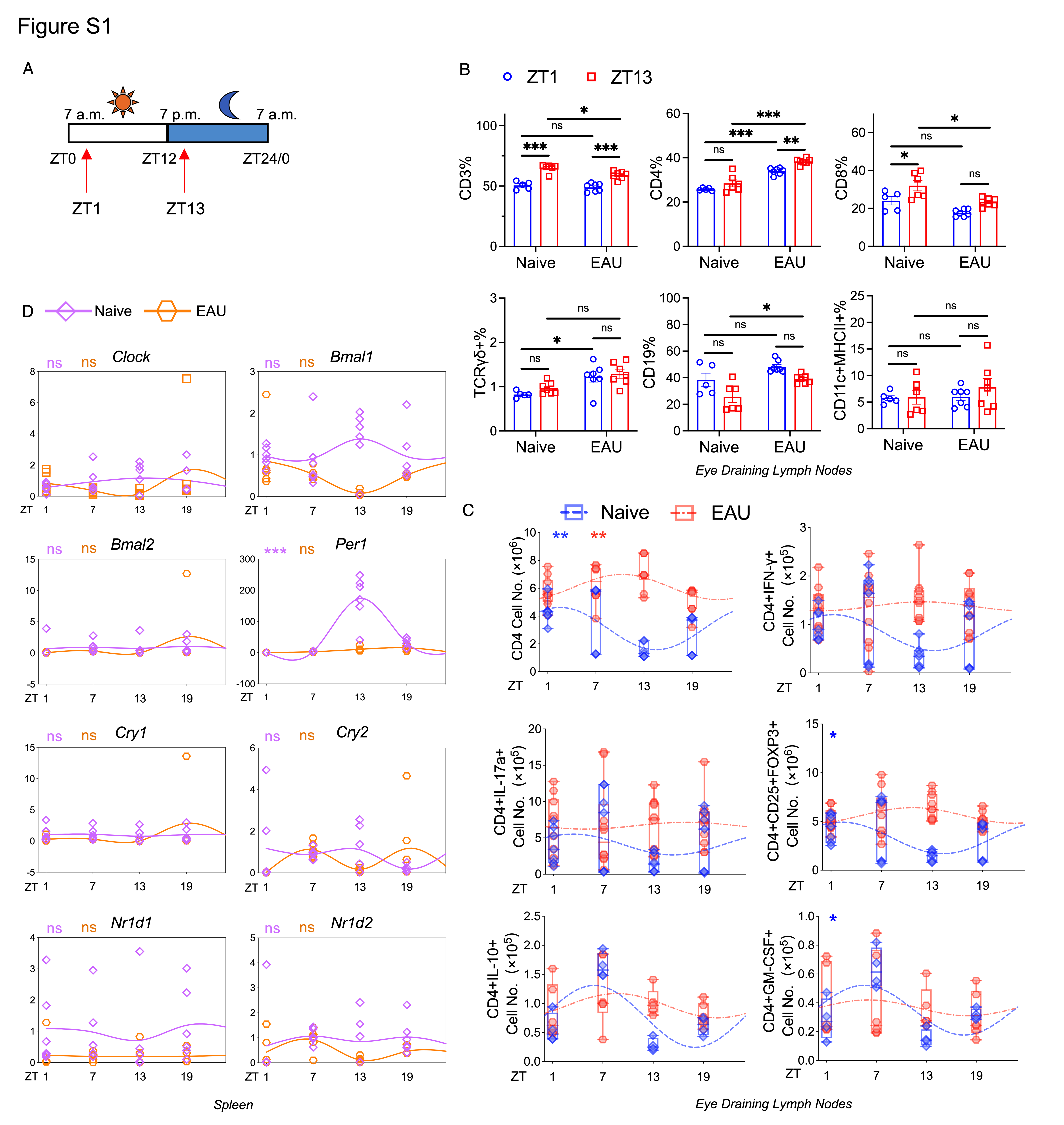
**

**Figure S1. Diurnal Rhythms in EAU and Normal Murine CD4+ T Cells.**

**(A)** Schematic diagram of Zeitgeber Time and the time points for sacrifice mice (ZT1, ZT13).
**(B)** Plots display the percentages at different time points of the indicated cell populations (CD3, CD4, CD8, TCRγδ, CD19, Dendritic Cells) in the eye-draining lymph cells of Naïve and EAU mice. N=5-7. Data was combined from two experiments.

**(C)** Plots display the cell number of indicated cell subsets (CD4+, Th1, Th17, Treg, IL-10+, GM-CSF+ CD4) at different time points and their fitted cosine curve in the eye-draining lymph cells of naïve and EAU mice (21 days post-immunization). N= 6. Data was combined from two experiments.

**(D)** A series of core-clock gene expressions in CD4+ T cells from the spleen of naïve and EAU mice were examined by RT-qPCR at different ZT times. N= 6. Data was combined from two experiments.

(B) Statistical significance was determined by 2-way ANOVA followed by Bonferroni test. (C-D) Cosine similarity analysis and statistical significance were determined by Consinor. Data are presented as mean ± SEM. * P＜0.05, ** P＜0.01，*** P＜0.001, ****P<0.0001.


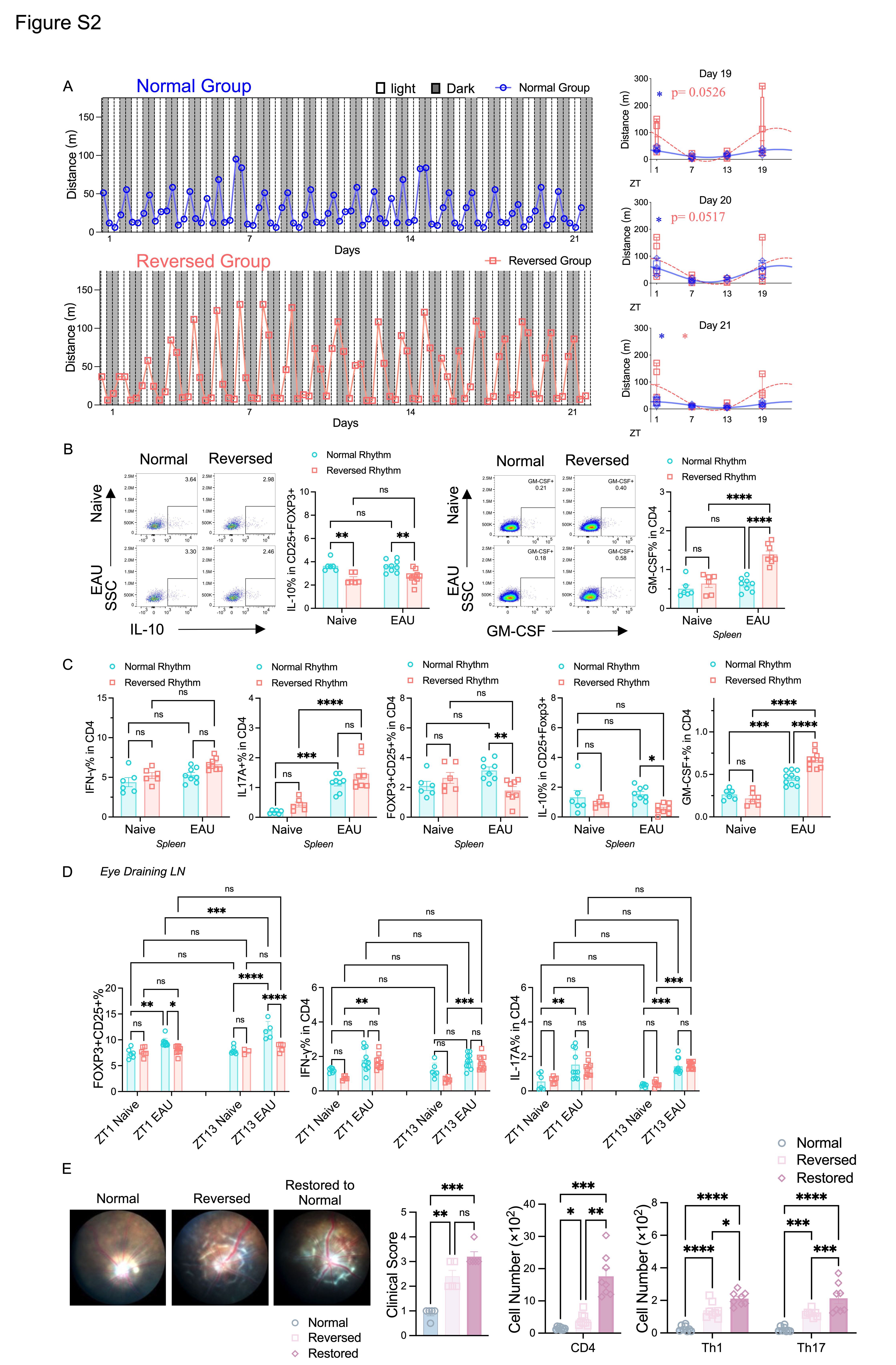


**Figure S2. Circadian rhythm disruption exacerbates EAU**.

**(A)** Mice from the Normal Rhythm Group and the Reversed Rhythm Group were placed under the camera respectively for 21 days and the traveling paths were recorded by ANY-maze. Cosinor statistical analysis were performed daily, and plots showed the travel paths on Day 19, 20, and 21. N= 4.

**(B)** Presentative FACS plots and bar graphs depicting the percentage of IL-10 and the GM-CSF in CD4+ T cells from the eye-draining lymph nodes of the Normal and the Reversed EAU mice at ZT 1 of Day 21 after immunization. N= 6-10. Data was combined from two experiments.

**(C)** Presentative FACS plots and bar graphs depicting the percentage of Th1 cells, Th17 cells, Tregs, IL-10+ cells, and GMCSF+ cells from the spleen of the Normal and the Reversed EAU mice at ZT 1 of Day 21 after immunization. N= 6-8. Data was combined from two experiments.

**(D)** Bar graphs showing the percentage of Treg, Th1, and Th17 cells in CD4+ T cells from the eye-draining lymph nodes of the Normal and the Reversed EAU mice at ZT 1 and ZT13 of Day 21 after immunization. N= 6-10. Data was combined from two experiments.

**(E-F)** Mice were divided into 3 groups normal (lights on at 7 a.m., lights off at 7 p.m.) or reversed (lights on at 7 p.m., lights off at 7 a.m.), restored (Reversed lighting for 10 days, and restored lighting for the left 11 days) cycles of light exposure, and all the mice were immunized for EAU. N= 8. Data was combined from two experiments. **(E)** Representative fundus images showing the fundoscopy from the indicated groups on Day 20 after immunization. Bar graphs showed the clinical scores of those EAU mice. **(F)** Bar graphs depicting the percentage of CD45+CD4+ cells, Th1 cells, and Th17 cells from the retina of the indicated group on Day 21 after immunization. N= 8. Data was combined from two experiments.

(B-F) Statistical significance was determined by 2-way ANOVA followed by Bonferroni test. Data are presented as mean ± SEM, with *p<0.05, **p<0.01, *** P＜0.001, ****P<0.0001.

**
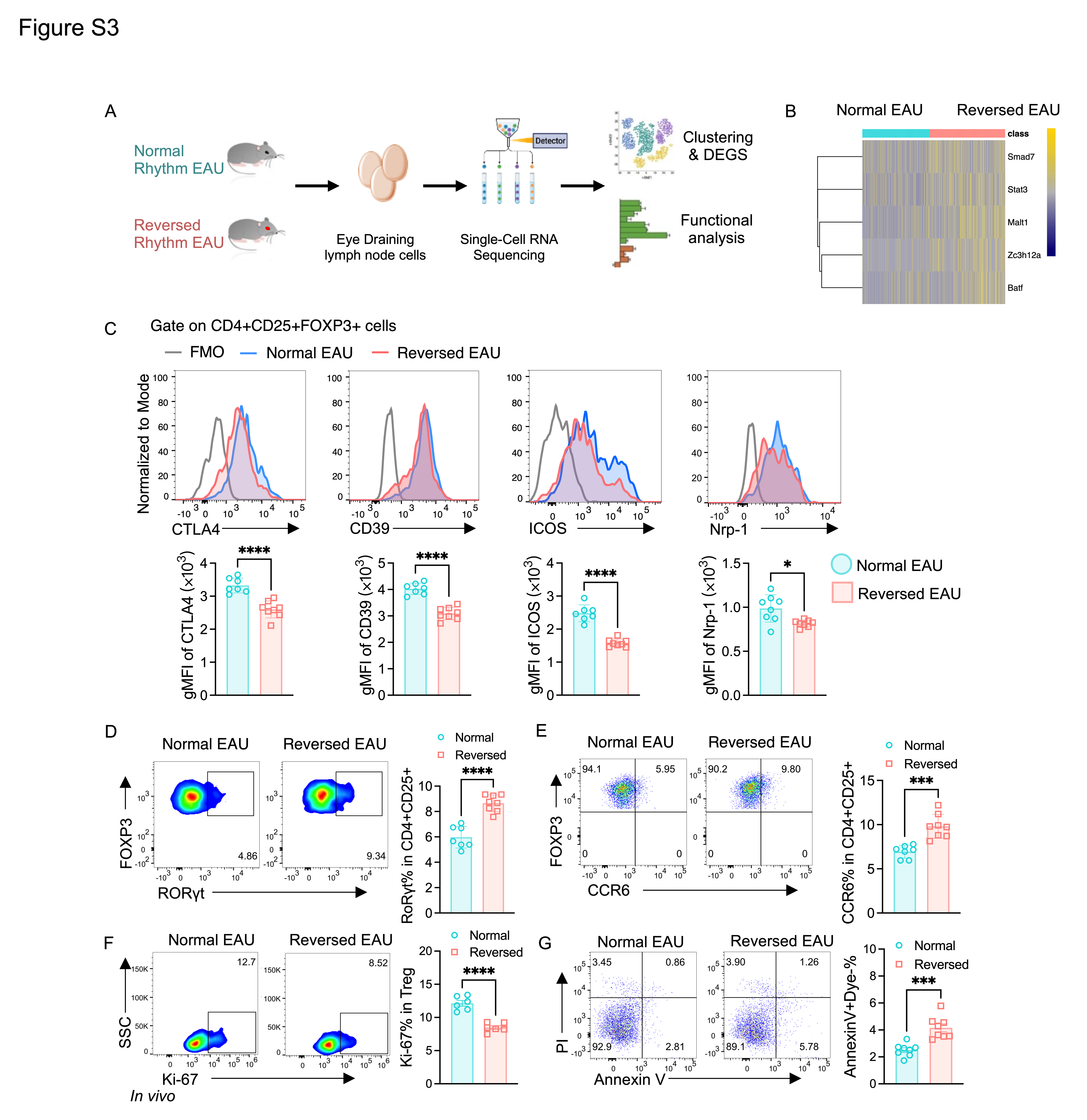
**

**Figure S3. Impairment of Treg stability and function due to circadian rhythm disruption.**

**(A)** Schematic diagram showing the workflow of single-cell RNA-seq from the normal and reversed group's eye-draining lymph nodes.

**(B)** Heatmap displaying inflammatory genes from DEGs between Tregs from the Normal and the Reversed Group.

**(C-G)** C57BL/6J mice were treated with normal (lights on at 7 a.m., lights off at 7 p.m.) or reversed (lights on at 7 p.m., lights off at 7 a.m.) cycles of light exposure. Mice were immunized with hIRBP1-20 on Day 0, and mice were sacrificed at ZT1 on Day 21. Representative FACS histograms and bar graphs presenting the expression of ICOS, CTLA4, CD39, and Nrp-1 **(C)**, RORγt+ **(D)**, CCR6+ **(E)**, Ki-67+ **(F)**, Dye-Annexin V+ **(G)** in the Treg cells from the Normal and Reversed group. N=7-8. Data were combined from two experiments.

Statistical significance was determined by Welch’s t-test. Data are presented as mean ± SEM, with *p<0.05, **p<0.01, ***p<0.001.

**
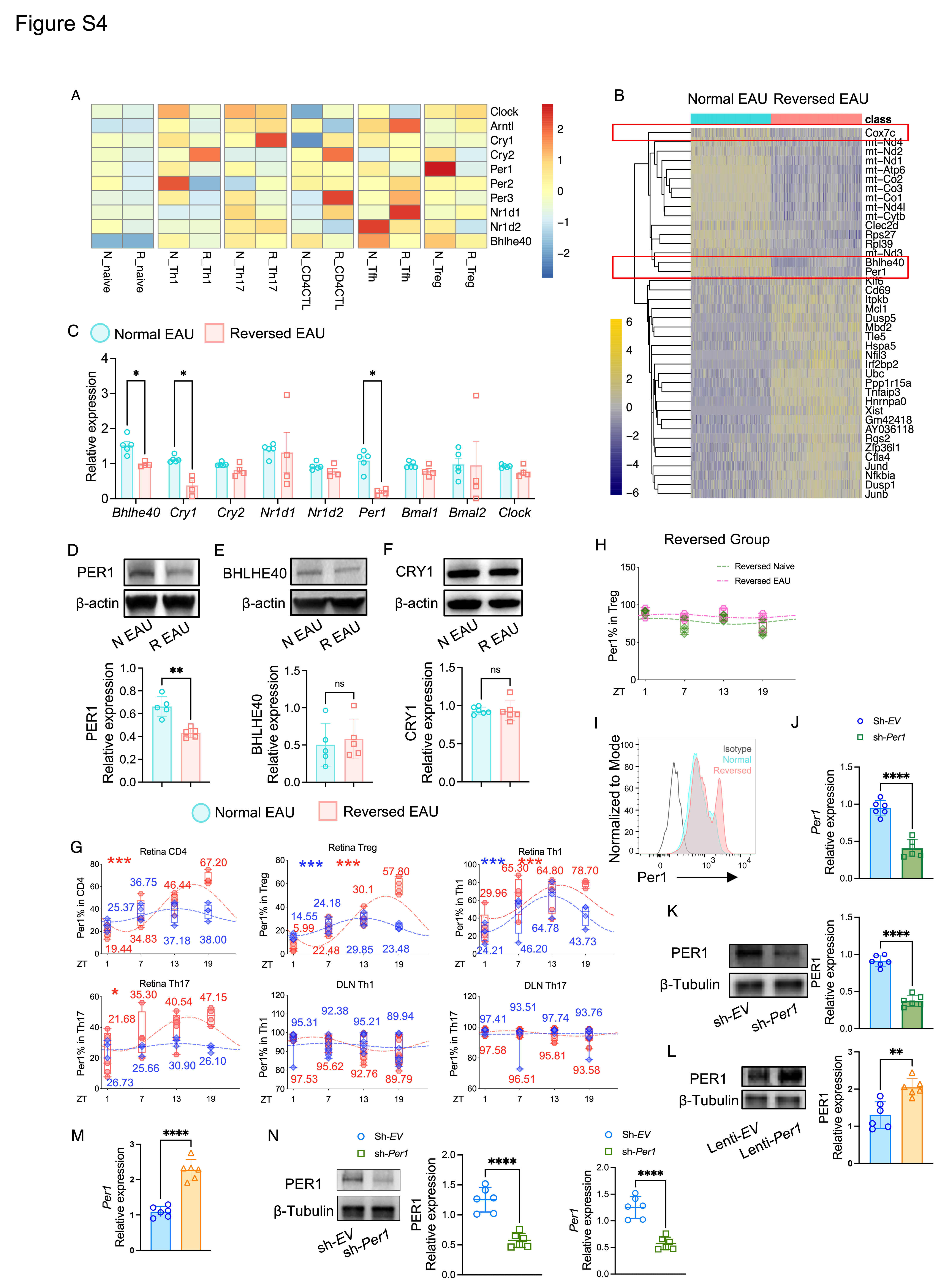
**

**Figure S4. Circadian rhythm regulation of Treg function via Per1.**

**(A)** Heatmap displaying the core clock genes from CD4+ T cell subsets between the Normal Rhythm Group and the Reversed Rhythm group.

**(B)** Heatmap displaying the FC top 40 genes from DEGs between the Tregs from the Normal Rhythm Group and the Reversed Rhythm group.

**(C)** Real-time qPCR was performed to validate the expression of core clock genes. N=4-5. Data was combined from two experiments.

**(D-F)** Protein expression of PER1, BHLHE40, and CRY1 was examined by Western blot in the eye-draining lymph nodes of the EAU mice from the Normal and the Reversed Groups. N=5-6. Data was combined from two experiments.

**(G)** Per1 percentages in CD4+ T cell subsets from the retina and eye-draining lymph nodes of naïve and EAU mice were examined by flow cytometry at different ZT times. Data was combined from two experiments. N=4-10.

**(H)** Per1 expressions in Tregs from naïve and EAU mice of the Reversed Group were examined by flow cytometry at different ZT times. N=4-5. Data was combined from two experiments.

**(I)** Representative histogram plots exhibit Per1 of Treg from the isotype, the normal and reversed group.

**(J)** RT-qPCR and **(K)** Western Blot data show the efficiency of sh-Per1 in the Treg cell. N=6. Data was combined from two experiments.

**(L)** Western Blot and RT-qPCR **(M)** Western Blot data show the efficiency of lenti-Per1 in the Treg cell. N=6. Data was combined from two experiments.

**(N)** Western Blot and RT-qPCR **(O)** Western Blot data show the efficiency of lenti-Per1 in the CD45.1/1 Treg cell. N=6. Data was combined from two experiments.

(C) Statistical significance was determined by 2-way ANOVA followed by Bonferroni test. (D-F, I-O) Statistical significance was determined by Welch’s t test. (H-I) Statistical significance was determined by Consinor. Data are presented as mean ± SEM, with *p<0.05, **p<0.01, *** P＜0.001, ****P<0.0001.


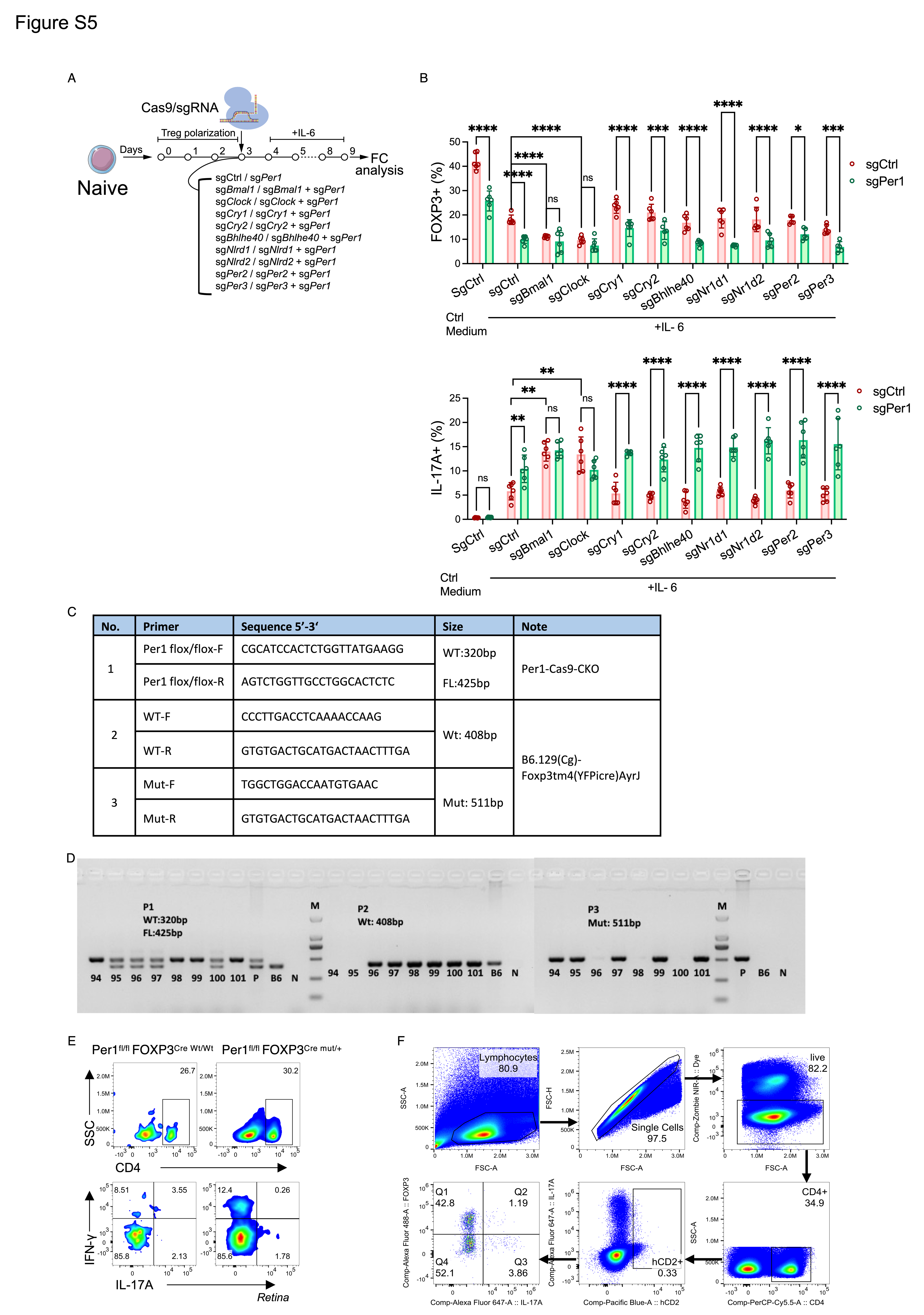


**Figure S5. Circadian rhythm regulation of Treg function via Per1.** Refer to Figure 4.

**(A-B)** Naïve CD4+ cells isolated from naïve C57B/L6j mice were treated under IL-2 (10ng/ml) and TGF-β (10ng/ml) in the presence of pre-coated anti-CD3 (1μg/ml) and soluble anti-CD28 (2μg/ml) for Treg polarization for 72 hours. Cells were transfected with each circadian gene Cas9/SgRNA with or without sg-Per1, and then cells were treated with IL-6 (50ng/mg) to induce unstable Treg for 5 days. **(A)** A diagram for the experiment. **(B)** Flow cytometry data showing the trans-differentiation of Treg towards Th17 cells with double knock-out of both Per1 and each circadian gene. N=6. Data were combined from two experiments.

**(C)** Table shows the primer for Per1 CKO genotyping.

**(D)** PCR image showed the identification of Per1 CKO. B6 represents a negative control, and N means blank control.

**(E)** Representative FACS plots depicting the percentage of CD45+CD4+ cells, Th1 (CD4+ IFN-γ+) cells, and Th17 (CD4+ IL-17A+) cells from the retina of the control and Per1 CKO EAU mice on Day 20 after immunization.

**(F)** FACS plots display the gating strategy of hCD2+ cells in Figure 4K.

(B) Statistical significance was determined by 2-way ANOVA followed by Bonferroni test. Data presented as means ± SEM, with *p<0.05, **p<0.01, ***P<0.001, ****P<0.0001.

**
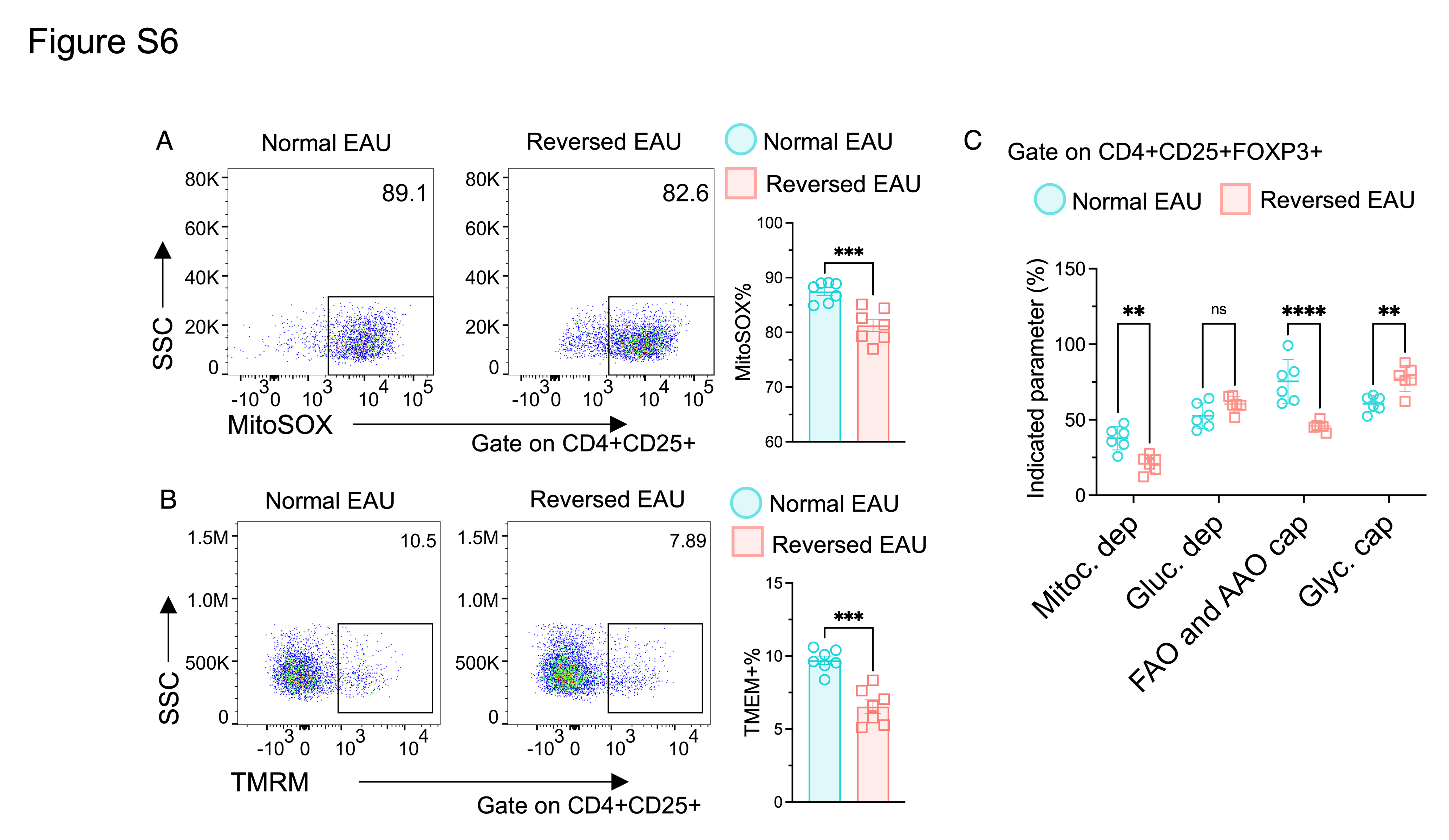
**

**Figure S6. Impaired mitochondrial function of regulatory T cells due to circadian disruption via Per1.** Refer to Fig. 5.

**(A-B)** Representative FACS plots and bar graphs show the MitoSOX and TMRM percentages in the Treg cells from the Normal Group and the Reversed Group. N=7. Data was combined from two experiments.

**(C)** SCENITHTM assay was performed for metabolic assessment of total lymphocytes from eye-draining lymph nodes. Culture medium, oligomycin (1 μM), 2-DG (100 mM), and oligomycin (1 μM) + 2-DG (100 mM) were added for 45 minutes in the respective wells. Puromycin was added to the co-culture for the last 30 minutes, and then all the cells were stained with anti-puromycin. Cells were gated on CD4+CD25+FOXP3+ to collect and calculate the MFI of puromycin. N= 6. Data combined from two experiments.

(A-B) Statistical significance was determined by Welch’s t-test. (C) Statistical significance was determined by 2-way ANOVA followed by Bonferroni test. Data presented as mean ± SEM, with *p<0.05, **p<0.01, ***p<0.001, ****p<0.0001.

**
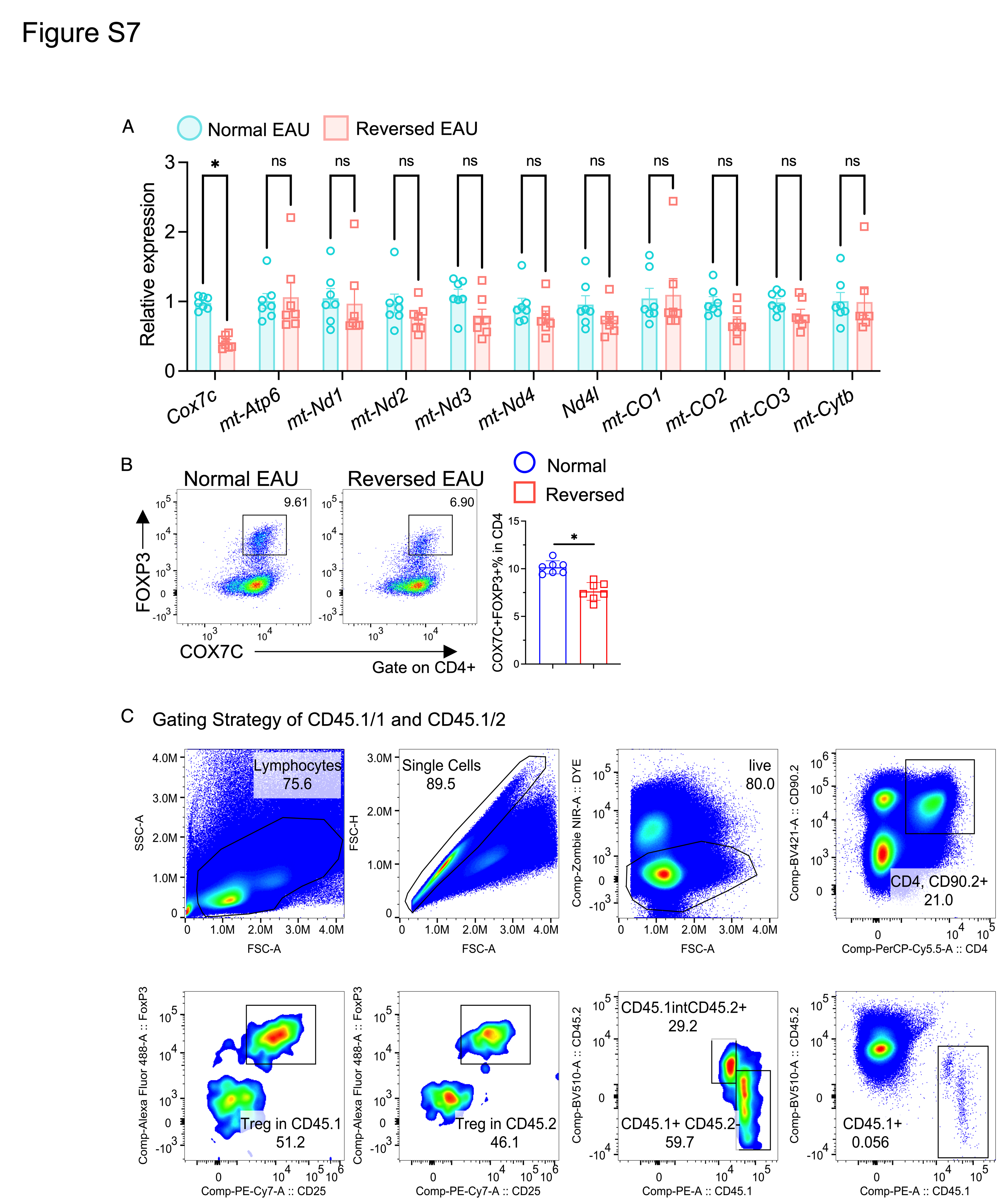
**

**Figure S7. Per1 modulates Treg stability through Cox7c.**Refer to Fig. 6.

**(A)** Real-time qPCR validation was performed to validate the expression of mitochondria-related genes in scRNA-seq. N=7. Data was combined from two experiments.

**(B)** Representative FACS plots and bar graphs displaying FOXP3+Cox7c+ percentage in the CD4+ T cell from the Normal Rhythm EAU and the Reversed Rhythm EAU mice. N=6. Data was combined from two experiments.

**(C)** FACS plots display the gating strategy of cells in Figure 6G.

(A) Statistical significance was determined by by 2-way ANOVA followed by Bonferroni test. (B) Statistical significance was determined Welch’s t-test. Data presented as mean ± SEM, with *p<0.05, **p<0.01, ***p<0.001, ****p<0.0001.

**Figure S8 and Table S1**

**Core clock protein expression oscillations in the major immune cell type of naïve and EAU mice**

| Cell Subset | cosinor_p | cosinor_q | Cell Subset | cosinor_p | cosinor_q |
| --- | --- | --- | --- | --- | --- |
| CD8 PER1 Naive | 0.060 | 0.275 | CD8 PER1 EAU | 0.058 | 0.275 |
| CD8 BMAL1 Naive | 0.397 | 0.657 | CD8 BMAL1 EAU | 0.417 | 0.657 |
| CD8 CRY1 Naive | 0.570 | 0.729 | CD8 CRY1 EAU | 0.886 | 0.978 |
| CD11b PER1 Naive | 0.156 | 0.410 | CD11b PER1 EAU | 0.025 | 0.250 |
| CD11b BMAL1 Naive | 0.785 | 0.901 | CD11b BMAL1 EAU | 0.452 | 0.657 |
| CD11b CRY1 Naive | 0.788 | 0.901 | CD11b CRY1 EAU | 0.443 | 0.657 |
| CD11C PER1 Naive | 0.196 | 0.449 | CD11C PER1 EAU | 0.129 | 0.404 |
| CD11C BMAL1 Naive | 0.936 | 0.999 | CD11C BMAL1 EAU | 0.037 | 0.250 |
| CD11C CRY1 Naive | 0.999 | 0.999 | CD11C CRY1 EAU | 0.008 | 0.250 |
| CD19 PER1 Naive | 0.072 | 0.289 | CD19 PER1 EAU | 0.028 | 0.250 |
| CD19 BMAL1 Naive | 0.555 | 0.729 | CD19 BMAL1 EAU | 0.990 | 0.999 |
| CD19 CRY1 Naive | 0.710 | 0.873 | CD19 CRY1 EAU | 0.391 | 0.657 |
| CD8 NR1D2 naive | 0.005 | 0.025 | CD8 NR1D2 EAU | 0.015 | 0.036 |
| CD8 CLOCK naive | 0.002 | 0.017 | CD8 CLOCK EAU | 0.072 | 0.085 |
| CD8 CRY2 naive | 0.013 | 0.036 | CD8 CRY2 EAU | 0.077 | 0.085 |
| CD8 NR1D1 naive | 0.002 | 0.017 | CD8 NR1D1 EAU | 0.125 | 0.125 |
| Macrophage NR1D2 naive | 0.038 | 0.057 | Macrophage NR1D2 EAU | 0.009 | 0.028 |
| Macrophage CLOCK naive | 0.000 | 0.008 | Macrophage CLOCK EAU | 0.008 | 0.028 |
| Macrophage CRY2 naive | 0.068 | 0.084 | Macrophage CRY2 EAU | 0.016 | 0.036 |
| Macrophage NR1D1 naive | 0.040 | 0.057 | Macrophage NR1D1 EAU | 0.017 | 0.036 |
| DC NR1D2 naive | 0.021 | 0.037 | DC NR1D2 EAU | 0.020 | 0.037 |
| DC CLOCK naive | 0.046 | 0.063 | DC CLOCK EAU | 0.000 | 0.008 |
| DC CRY2 naive | 0.103 | 0.107 | DC CRY2 EAU | 0.077 | 0.085 |
| DC NR1D1 naive | 0.047 | 0.063 | DC NR1D1 EAU | 0.103 | 0.107 |
| CD19 NR1D2 naive | 0.004 | 0.024 | CD19 NR1D2 EAU | 0.014 | 0.036 |
| CD19 CLOCK naive | 0.019 | 0.037 | CD19 CLOCK EAU | 0.006 | 0.028 |
| CD19 CRY2 naive | 0.022 | 0.037 | CD19 CRY2 EAU | 0.036 | 0.057 |
| CD19 NR1D1 naive | 0.008 | 0.028 | CD19 NR1D1 EAU | 0.050 | 0.065 |

Plots exhibit the expressions of core clock proteins measured by Flow Cytometer. There are 4-time points for 24h (ZT1, ZT7, ZT13, ZT19). N=6-10. Tables exhibit the p and q value for each core clock protein in each subset of EAU and naïve mice. Data was combined from 2 experiments. Statistics were calculated by Cosinor. * q < 0.05, ** q< 0.01.

**Table S2 - Plasmid for clock gene knockdown**

| Name | Sequence |
| --- | --- |
| mClock-sgRNA1 | TCCTGGTAACGCGAGAAAGA |
| mClock-sgRNA2 | TCCATCTTTCTCGCGTTACC |
| mBmal1-sgRNA1 | TCAGATTGAAAAGAGGCGTC |
| mBmal1-sgRNA2 | TAGATAAACTCACCGTGCTA |
| mCry1-sgRNA1 | TTCGCCGGCTCTTCCAACGT |
| mCry1-sgRNA2 | ATCCTCGACCCCTGGTTCGC |
| Cry2-sgRNA1 | ACGGTCCCCGCGCAATCGAT |
| Cry2-sgRNA2 | GTCCGCGCCCATCGATTGCG |
| Per1-sgRNA1 | GCCCCTGGACTCGGGTCCGT |
| Per1-sgRNA2 | CCCCTAGAAGGGGCCGATGG |
| Per2-sgRNA1 | GCAGTGACTGCGACGACAAT |
| Per2-sgRNA2 | GGAGCAGTTCTCGTTTCCGC |
| Per3-sgRNA1 | AAAAGTATTTCCCAGCCGAG |
| Per3-sgRNA2 | GCTTAGTGTGCCTCTCGGCT |
| Nr1d1-sgRNA1 | CTAGTGGCTCCTCCCCGAGC |
| Nr1d1-sgRNA2 | GACTCCGGGCTGGTCCGGCT |
| Nr1d2-sgRNA1 | GCTGATATCTCTAGCATCGA |
| Nr1d2-sgRNA2 | AGCGTTCTTGGGATTGCCGT |
| Bhlhe40-sgRNA1 | AAACTTACAAACTGCCGCAC |
| Bhlhe40-sgRNA2 | GGGCAATGCACTCGTTAATC |
| hPER1-sgRNA1 | GGCCACGCTGCAGTACGCAC |
| hPER1-sgRNA2 | TCGACTGCCGCCAGAGCGCC |
| mper1-shRNA-1 | CCGG-TTCGTGTTGGGTCGCCATAAA-CTCGAG-TTTATGGCGACCCAACACGAA-TTTTTT |
| mper1-shRNA-2 | CCGG-GGTGCTCCCTAACTATCTATT-CTCGAG-AATAGATAGTTAGGGAGCACC-TTTTTT |
| \| mCox7c-shRNA-1 \| \| --- \| | \| CCGG-CCGCACCTTTCTTTATAGTAACTCGAGTTACTATAAAGAAAGGTGCGG-TTTTTT \| \| --- \| |
| mCox7c-shRNA-2 | CCGG-GCCGCACCTTTCTTTATAGTA-CTCGAG-TACTATAAAGAAAGGTGCGGC-TTTTTT |
| mCox7c-shRNA-3 | CCGG-GCTATGATGACCGTGTACTTT-CTCGAG-AAAGTACACGGTCATCATAGC-TTTTTT |
| mCox7c-shRNA-4 | CCGG-CGTGTACTTTGGATCTGGGTT-CTCGAG-AACCCAGATCCAAAGTACACG-TTTTTT |
| NC(4106-81) | CCGG-GATTCTCCGAACGTGTCACGT-CTCGAG-ACGTGACACGTTCGGAGAATC-TTTTTT |

**Table S3 - Antibody list**

| Target | Fluorochrome | Supplier | Cat no. | RRID |
| --- | --- | --- | --- | --- |
| Anti-Mouse CD45 | BV510 | BioLegend | 103137 | AB_2561392 |
| Anti-Mouse CD3 | PE | BioLegend | 100206 | AB_312663 |
| Anti-Mouse CD4 | PerCP-Cy5.5 | BioLegend | 100434 | AB_893324 |
| Anti-Mouse CD8 | BV785 | BioLegend | 100749 | AB_11218801 |
| Anti-Mouse CD25 | PE-Cyanine7 | BioLegend | 102016 | AB_312865 |
| Anti-Mouse TCRγδ | APC | eBioscience, Thermo Fisher | 17-5711-82 | AB_842756 |
| Anti-mouse CD45.1 | PE | BioLegend | 110707 | AB_313496 |
| Anti-mouse CD45.2 | BV510 | BioLegend | 109837 | AB_2650900 |
| Anti-mouse CCR6 | BV605 | BioLegend | 129819 | AB_2562513 |
| Anti-Human/mouse/Rat ICOS | BV785 | BioLegend | 313534 | AB_2629728 |
| Anti-mouse CTLA4 | PE | eBioscience, Thermo Fisher | 12-1529-42 | AB_10805626 |
| Anti-mouse Nrp-1 | BV421 | BioLegend | 145209 | AB_2562358 |
| Anti-mouse CD39 | APC | BioLegend | 143810 | AB_2750320 |
| anti-Annexin V antibody | PE | BD Bioscience | 556421 | AB_2869071 |
| Anti-mouse CD19 | BV785 | BioLegend | 115543 | AB_11218994 |
| Anti-human/mouse CD11b | PE | BioLegend | 101208 | AB_312790 |
| Anti-mouse CD11c | Percp-cy5.5 | BioLegend | 117327 | AB_2129641 |
| Anti-mouse MHCII | BV605 | BioLegend | 107639 | AB_2565894 |
| Anti-mouse F4/80 | FITC | BioLegend | 123108 | AB_893500 |
| Anti-mouse IFN-γ | BV785 | BioLegend | 505838 | AB_2629667 |
| Anti-mouse IL-17A | BV650 | BioLegend | 506930 | AB_2686975 |
| Anti-Human/mouse FOXP3 | FITC | eBioscience, Thermo Fisher | 11-5773-82 | AB_465243 |
| Anti-mouse GM-CSF | PE-CY7 | BioLegend | 505412 | AB_2721681 |
| Anti-mouse IL-10 | BV421 | BioLegend | 505022 | AB_2563240 |
| Anti-mouse RORγt | BV421 | BD Bioscience | 562894 | AB_2687545 |
| Anti-mouse Ki67 | BV605 | BioLegend | 652413 | AB_2562664 |
| Anti-Mouse/Human-CLOCK | FITC | Bioss | bs-20500R-FITC | N/A |
| Anti-Mouse/Human-CRY2 | PE | Bioss | bs-11447R-PE | N/A |
| Anti-Mouse/Human-NR1D1 | PE-CY7 | Bioss | bs-3563R-PE-Cy7 | AB_11071939 |
| Anti-Mouse/Human-PER1 | AF647 | Bioss | bs-2350R-AF647 | AB_10857437 |
| Anti-Mouse/Human-CRY1 | PE-CY5 | Bioss | bs-11441R-PE-Cy5 | N/A |
| Anti-Mouse/Human-BMAL1 | PE | Bioss | bs-3750R-PE | AB_11047865 |
| Anti-Mouse/Human-NR1D2 | APC | Bioss | bs-20223R-APC | N/A |
| Anti-Mouse/Human-COX7C | PE-CY7 | Bioss | bs-14016R-PE-Cy7 | N/A |
| Anti-Human CD4 | PE | BioLegend | 300508 | AB_314075 |
| Anti-Human CD25 | BV421 | BioLegend | 302630 | AB_10896914 |
| Anti-Human IFN-γ | AF647 | BioLegend | 502542 | AB_11219192 |
| Anti-Human IL-17A | BV650 | BD Bioscience | 563746 | AB_2738402 |
| Zombie NIR Dye | N/A | BioLegend | 423106 | N/A |

**Table S4 - Patients Information**

| Patients ID | Age (Year) | Gender | Disease Duration | Result |
| --- | --- | --- | --- | --- |
| 1 | 55 | Male | BD Active | Figure 7 |
| 2 | 32 | Female | BD Active | Figure 7 |
| 3 | 24 | Male | BD Active | Figure 7 |
| 4 | 22 | Female | BD Active | Figure 7 |
| 5 | 18 | Male | BD Active | Figure 7 |
| 6 | 52 | Male | BD Inactive | Figure 7 |
| 7 | 46 | Male | BD Inactive | Figure 7 |
| 8 | 23 | Male | BD Inactive | Figure 7 |
| 9 | 20 | Female | BD Inactive | Figure 7 |
| 10 | 18 | Female | BD Inactive | Figure 7 |
